# Structural and kinetic insights into the stereospecific oxidation of *R*-2,3-dihydroxypropanesulfonate by DHPS-3-dehydrogenase from *Cupriavidus pinatubonensis*†

**DOI:** 10.1039/d4sc05114a

**Published:** 2024-09-09

**Authors:** Laura Burchill, Arashdeep Kaur, Artur Nastasovici, Mihwa Lee, Spencer J. Williams

**Affiliations:** a School of Chemistry, Bio21 Molecular Science and Biotechnology Institute, University of Melbourne Parkville Victoria 3010 Australia mihwa.lee@unimelb.edu.au sjwill@unimelb.edu.au

## Abstract

2,3-Dihydroxypropanesulfonate (DHPS) and sulfolactate (SL) are environmentally important organosulfur compounds that play key roles as metabolic currencies in the sulfur cycle. Despite their prevalence, the pathways governing DHPS and SL production remain poorly understood. Here, we study DHPS-3-dehydrogenase from *Cupriavidus pinatubonensis* (*Cp*HpsN), a bacterium capable of utilizing DHPS as a sole carbon source. Kinetic analysis of *Cp*HpsN reveals a strict preference for *R*-DHPS, catalyzing its 4-electron oxidation to *R*-SL, with high specificity for NAD^+^ over NADP^+^. The 3D structure of *Cp*HpsN in complex with Zn^2+^, NADH and *R*-SL, elucidated through X-ray crystallography, reveals a fold akin to bacterial and plant histidinol dehydrogenases with similar coordination geometry around the octahedral Zn^2+^ centre and involving the sulfonate group as a ligand. A key residue, His126, distinguishes DHPS dehydrogenases from histidinol dehydrogenases, by structural recognition of the sulfonate substrate of the former. Site-directed mutagenesis pinpoints Glu318, His319, and Asp352 as active-site residues important for the catalytic activity of *Cp*HpsN. Taxonomic and pathway distribution analysis reveals the prevalence of HpsN homologues within different pathways of DHPS catabolism and across bacterial classes including Alpha-, Beta-, Gamma-, and Deltaproteobacteria and Desulfobacteria, emphasizing its importance in the biogeochemical sulfur cycle.

## Abstract 2

Structural and kinetic analyses of HpsN from *Cupriavidus pinatubonensis* that catalyses the stereospecific oxidation of an important organosulfur compound, *R*-2,3-dihydroxypropanesulfonate are presented.

## Graphical abstract

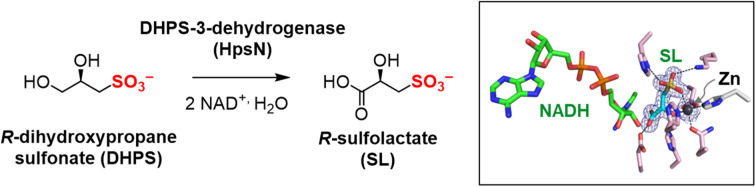

## 1 Introduction

2,3-Dihydroxypropanesulfonate (DHPS) and sulfolactate (SL) play significant roles as organosulfonate compounds within the Earth's biosulfur cycle.^1^ These compounds are the products of sulfoglycolysis of SQ,^2,3^ the sugar headgroup of sulfolipid (sulfoquinovosyl diacylglycerol, SQDG). SQDG is synthesized by all photosynthetic organisms, including plants, algae, and cyanobacteria.^4,5^ Notably, DHPS is also produced by marine diatoms and coccolithophores,^6,7^ and can be detected in seawater during algal blooms,^8^ although the biochemical pathway(s) for its formation in these contexts is still not fully understood.^6^ Similarly, SL is produced by sporulating *Bacillus subtilis* but the pathway(s) for its production remain undefined.^9^ The global annual production of SQ is estimated as 10^10^ tonnes.^5^ Given that SQ and DHPS can be metabolized into SL, it is reasonable to assume that SL is also synthesized on a comparable scale.^1^ The metabolism of DHPS, *via* SL, connects organosulfonate metabolites with secondary bacterial metabolites such as tropodithietic acid, an algal protecting bacteriocide.^10^

**Fig. 1:**
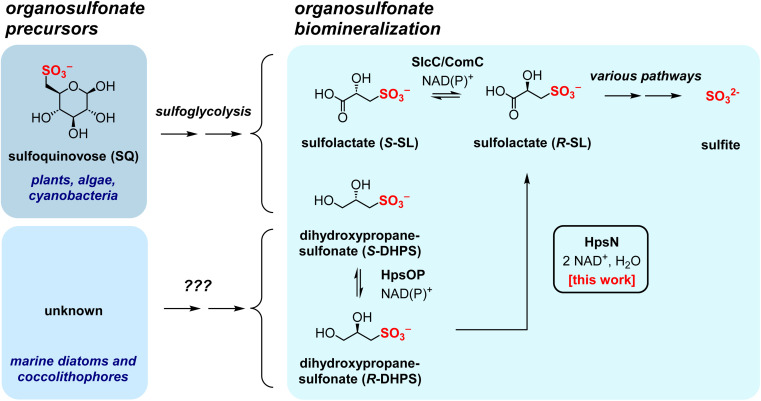
Pathways for formation of 2,3-dihydroxypropanesulfonate (DHPS) and sulfolactate (SL), oxidation to SL catalyzed by DHPS-3-dehydrogenase (HpsN), and biomineralization to sulfite. DHPS is produced through sulfoglycolysis or through unknown eukaryotic pathways. SL can also be produced by sulfoglycolysis and by oxidation of DHPS. In this work we show that DHPS-3-dehydrogenase HpsN from *Cupriavidus pinatubonensis* acts selectively on *R*-DHPS to give *R*-SL.

**Fig. 2:**
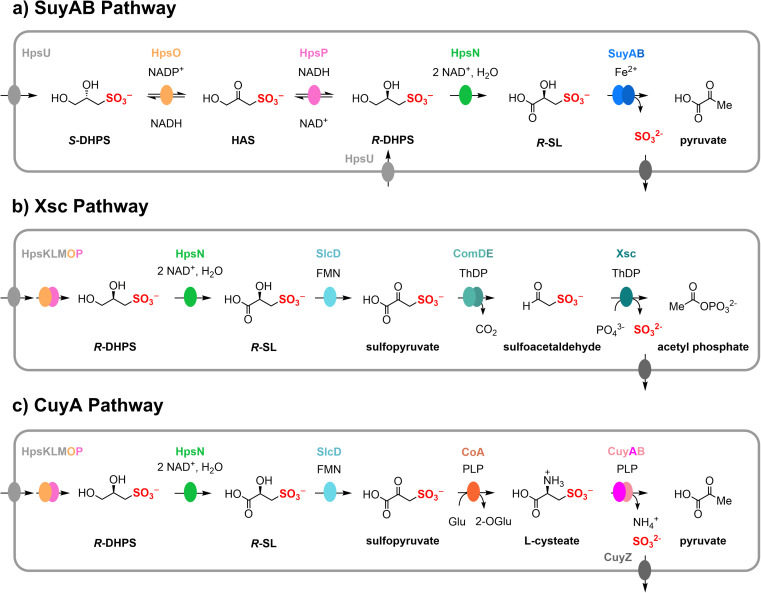
Various DHPS biomineralization pathways *via* SL as an intermediate, formed through the action of DHPS-3-dehydrogenase (HpsN). (a) Fe^2+^-dependent SL lyase (SuyAB) pathway. (b) Thiamine diphosphate-dependent sufoacetaldehyde acetyltransferase (Xsc) pathway. (c) PLP-dependent cysteate lyase (CuyA) pathway.

Bacterial sulfoglycolysis of SQ forms *S*-SL and *S*-DHPS (Fig. 1).^11–15^ The individual enantiomers of DHPS are produced by various marine diatoms and coccolithophores, with some coccolithophores producing both molecular antipodes.^7^ A pathway for the interconversion of DHPS enantiomers in *Cupriavidus pinatubonensis* has been proposed involving a two-component system of HpsO and HpsP, which are NAD(P)^+^-dependent DHPS-2-dehydrogenases.^16^ Oxidation of DHPS by NAD^+^-dependent DHPS-3-dehydrogenase HpsN gives SL.^16^ A related pathway has been proposed in *Roseobacter pomeroyi*.^7^ The enantiomers of SL can be interconverted by the NAD(P)^+^-dependent SL-2-dehydrogenases SlcC and ComC, *via* sulfopyruvate.^17^ SL is a substrate for various metabolic pathways, as shown in Fig. 2. One pathway involves the Fe^2+^-dependent SL lyase SuyAB, which catalyzes the elimination of sulfite from SL to give pyruvate.^16–18^ A second pathway involves the oxidation of SL to sulfopyruvate (catalyzed by SlcC or ComC), which allows for decarboxylation, catalyzed by ComDE, to give sulfoacetaldehyde. Sulfoacetaldehyde is subsequently converted to acetyl phosphate and sulfite through the catalytic action of thiamine diphosphate (ThDP)-dependent sulfoacetaldehyde acetyltransferase Xsc.^19^ A third pathway entails the reductive amination of sulfopyruvate with glutarate, affording l-cysteate. CuyB is a racemase that interconverts l-cysteate with d-cysteate, with the latter being the preferred substrate for the pyridoxal 5′-phosphate (PLP)-dependent CuyA, leading to the formation of pyruvate, ammonia and sulfite.^19,20^

All of the above sulfolysis pathways feature DHPS dehydrogenase HpsN. This enzyme was originally identified in *Cupriavidus pinatubonensis* JMP134, a bacterium that can grow on DHPS as sole carbon source.^16^ HpsN, when purified and studied was a homodimer that converted racemic DHPS to SL using NAD^+^ as a cofactor. HpsN was predicted to act specifically on *R*-DHPS to generate *R*-SL,^16^ with recent experimental evidence confirming this prediction.^7^ In *C. pinatubonensis*, the gene responsible for HpsN lies within the *hpsRNOUPsuyAB* gene cluster. This cluster also encodes transcriptional regulator HpsR, DHPS-2-oxidoreductases HpsO and HpsP, a major facilitator superfamily uptake system HpsU, and SL lyase SuyAB. Notably, HpsN is sequence- and structurally-related^7^ to histidinol dehydrogenase (HisD), a Zn^2+^ and NAD^+^ dependent enzyme that oxidizes l-histidinol to histidine. While HisD has been extensively investigated,^21–23^ very little is currently known about the molecular mechanisms underlying catalysis by DHPS-3-dehydrogenase HpsN.

In this study, we present kinetic and structural analyses of DHPS-3-dehydrogenase HpsN from *C. pinatubonensis.* We conducted Michaelis–Menten kinetics on individual enantiomers of DHPS, revealing a marked preference for *R*-DHPS, and high specificity for NAD^+^*versus* NADP^+^. We show that HpsN catalyzes oxidation of *R*-DHPS to afford SL, and that SLA is also a substrate for the enzyme. Notably, HpsN is highly selective for sulfonated substrates and does not exhibit any activity towards l-histidinol or the structural analogue glycerol-1-phosphate. Furthermore, we report the 3D structure of HpsN, determined by X-ray crystallography, in complex with NADH and *R*-SL. The structure defines the coordination environment about the Zn^2+^ centre, pinpoints a key residue involved in recognizing the sulfonate group, and identifies possible catalytic residues, which we investigate by site-directed mutagenesis. Lastly, we explore the taxonomic distribution of DHPS-3-dehydrogenases within DHPS degradation pathways, shedding light on the ecological distribution of DHPS metabolic pathways.

## 2 Results

### 2.1. HpsN is a metalloenzyme that is selective for *R*-DHPS and can utilize SLA as a substrate

We designed the codon harmonized gene for *C. pinatubonensis* HpsN (WP_011295860.1) for expression in *E. coli*. Subsequently, we expressed and purified the recombinant protein, *Cp*HpsN. As HisD is a Zn^2+^-dependent enzyme, *Cp*HpsN was incubated with EDTA, purified by size exclusion chromatography, then reconstituted with five-fold molar excess of ZnCl_2_. Mass photometry analysis of 50 nM Zn^2+^-loaded *Cp*HpsN (calculated monomer molecular mass of 46.9 kDa) showed a single peak at 90 kDa, indicating a dimer in solution (Fig. S1†). All subsequent experiments used this Zn^2+^ reconstituted protein.

**Fig. 3:**
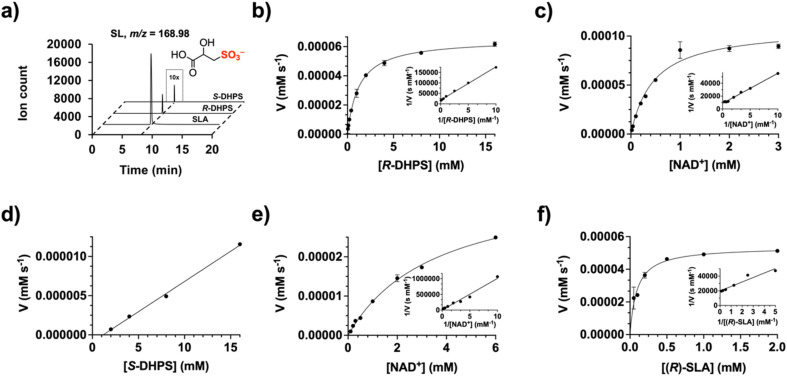
*Cp*HpsN produces SL from *R*- and *S*-DHPS: kinetic analysis and product studies. (a) HPLC mass spectrometry (triple quadrupole, QqQ) chromatograms showing *Cp*HpsN (DHPS-3-dehydrogenase) catalyzed conversion of *R*-DHPS, *S*-DHPS and SLA to SL at time = 4 h. (b)–(f) Kinetic studies and double reciprocal plots for *Cp*HpsN. Reactions were conducted in 100 mM Bis–Tris Propane (BTP) buffer (pH = 8.0) with 200 mM NaCl and 50 μM ZnCl_2_. All data shown is the mean of reaction rates (performed in triplicate). Error bars show standard error mean. (b) Michaelis–Menten and Lineweaver–Burk (inset) plots for *Cp*HpsN catalyzed oxidation of *R*-DHPS under pseudo first-order conditions of [NAD^+^] = 0.30 mM, and (c) [*R*-DHPS] = 8.0 mM. (d) Michaelis–Menten and Lineweaver–Burk (inset) plots for oxidation of *S*-DHPS by *Cp*HpsN under pseudo first-order conditions of [NAD^+^] = 0.30 mM and (e) [*S*-DHPS] = 8.0 mM. (f) Michaelis–Menten and Lineweaver–Burk (inset) plots for oxidation of *R*-SLA by *Cp*HpsN under pseudo first-order conditions of [NAD^+^] = 0.30 mM.

We initially assessed whether *Cp*HpsN could catalyze the oxidation of the two enantiomers of DHPS. Reaction mixtures of *Cp*HpsN, NAD^+^ and *R*- or *S*-DHPS were analyzed by liquid chromatography-mass spectrometry (LCMS) with a triple quadrupole (QqQ) mass spectrometer in product ion mode. The SL peak formed from *R*-DHPS was much larger than that formed from *S*-DHPS, indicating a clear preference of *Cp*HpsN for *R*-DHPS (Fig. 3a). As HpsN catalyzes a four-electron oxidation, we tested whether the proposed intermediate, sulfolactaldehyde (SLA) could serve as a substrate for *Cp*HpsN. LCMS analysis demonstrated formation of a substantial peak for SL, suggesting that this is an even better substrate for this enzyme (Fig. 3a).

While it is well-established that histidinol dehydrogenases are Zn^2+^-dependent metalloenzymes, the metal dependency of HpsN has not been comprehensively studied. In the presence of 1 mM EDTA, the activity of Zn^2+^-loaded *Cp*HpsN was reduced 250 000-fold. We then dialysed the EDTA-treated *Cp*HpsN, to obtain demetallated *Cp*HpsN, and added various divalent metals to study the reconstitution of activity using [NAD^+^] = 0.3 mM and [*R*-DHPS] = 8.0 mM. As mentioned above, demetallated HpsN lost essentially all activity, establishing it as a metalloenzyme (Fig. S2†). All divalent metals led to at least a partial recovery of activity. Maximum activity was observed with Co^2+^, followed closely by Zn^2+^ > Mn^2+^ > Mg^2+^ ≈ Ca^2+^ ≈ Ba^2+^ ≈ Cu^2+^ > Ni^2+^. Based on the close relationship with Zn^2+^-dependent histidinol dehydrogenases, the high intracellular concentration of Zn^2+^, and the rarity of cobalt-metalloenzymes, we continued to study the Zn^2+^ form of *Cp*HpsN.

### 2.2. Kinetic analysis of HpsN

To quantify the preference of *Cp*HpsN for the individual DHPS enantiomers and SLA, as well as for the cofactors NAD^+^*versus* NADP^+^, we conducted kinetic studies. Reaction rates were continuously monitored by observing the reduction of NAD(P)^+^ to NAD(P)H using a UV/visible spectrometer. Maximum activity was observed at pH 8, which was used for all subsequent analysis (Fig. S3†). Apparent Michaelis–Menten parameters were measured for *R*- and *S*-DHPS, and for NAD^+^ under pseudo first-order conditions, where one substrate was held at a constant concentration while that of the other was varied (Fig. 3 and Table 1). At an NAD^+^ concentration of 0.3 mM, the pseudo first-order parameters for *R*-DHPS were as follows: *k*^app^_cat_ = 0.97 s^−1^, *K*^app^_M_ = 1.3 mM and (*k*_cat_/*K*_M_)^app^ = 0.75 mM^−1^ s^−1^, while for *S*-DHPS we calculated: (*k*_cat_/*K*_M_)^app^ = 7.7 × 10^−7^ mM^−1^ s^−1^; in the latter case the enzyme did not display saturation so *k*^app^_cat_ and *K*^app^_M_ values could not be determined. Therefore, in terms of (*k*_cat_/*K*_M_)^app^ values, HpsN demonstrates a 10^6^-fold preference for *R*-DHPS. We also determined Michaelis–Menten kinetics for NAD^+^ while keeping concentrations of *R*- and *S*-DHPS constant at 8.0 mM. This analysis revealed a 100-fold higher (*k*_cat_/*K*_M_)^app^ value for NAD^+^ when *R*-DHPS was the substrate, arising from approximately 10-fold increases in the *k*^app^_cat_ and *K*^app^_M_ values. Additionally, when *R*-DHPS was selected as the preferred substrate (at 8.0 mM), no enzymatic activity was observed with NADP^+^. Hence, HpsN relies strictly upon NAD^+^ as cofactor.

**Table 1:** Kinetic analysis of assorted substrates for DHPS-3-dehydrogenase HpsN from *Cupriavidus pinatubonensis*^a^

Entry	Variable substrate	Constant substrate	Concentration (mM)	*k* ^app^_cat_ (s^−1^)	*K* ^app^_M_ (mM)	(*k*_cat_/*K*_M_)^app^ (mM^−1^ s^−1^)
1	*R*-DHPS	NAD^+^	0.30	0.97 ± 0.03	1.3 ± 0.15	0.75 ± 0.20
2	*S*-DHPS	NAD^+^	0.30	—	—	7.7 × 10^^−^7^^b^
3	NAD^+^	*R*-DHPS	8.00	1.60 ± 0.11	0.47 ± 0.10	3.4 ± 1.10
4	*R*-SLA	NAD^+^	0.30	1.58 ± 0.05	0.36 ± 0.06	8.6 ± 0.83
5	NAD^+^	*S*-DHPS	8.00	—	—	0.048^b^
6	*R*-DHPS	NADP^+^	0.30	ND	ND	ND
7	Glycerol phosphate	NAD^+^	0.30	ND	ND	ND
8	l-Histidinol	NAD^+^	0.30	ND	ND	ND

a ND, no activity detected.

b Saturation not achieved.

Our HPLC analysis indicated that SLA is a more favorable substrate than *R*-DHPS. Since our synthetic SLA is racemic, we initially examined its consumption. The incubation of a solution of racemic SLA (0.3 mM) with *Cp*HpsN and excess NAD^+^ (8 mM) gave a progress curve that suggested complete reaction after 2 h (Fig. S4†). Addition of more *Cp*HpsN did not result in further reaction. Using the extinction coefficient for NAD^+^, we calculate that 48 ± 1% of the SLA was consumed. This finding implies that *Cp*HpsN is stereospecific for *R*-SLA, and thus for kinetic analysis we adjusted the concentration for only this stereoisomer (*i.e.* [SLA]/2). At 0.3 mM NAD^+^, the pseudo first-order parameters for *R*-SLA were: *k*^app^_cat_ = 1.58 s^−1^, *K*^app^_M_ = 0.36 mM and (*k*_cat_/*K*_M_)^app^ = 8.6 mM^−1^ s^−1^. Therefore, *R*-SLA is approximately 12-fold more efficient as a substrate for *Cp*HpsN than *R*-DHPS in terms of (*k*_cat_/*K*_M_)^app^ value, mainly caused by a 3.6-fold lower *K*^app^_M_ value. This should be considered a lower estimate of the greater efficiency of *R*-SLA, as the enantiomer *S*-SLA may act as a competitive inhibitor.

Next, we investigated if *Cp*HpsN has activity on other non-sulfonated substrates. Glycerol-1-phosphate, which is structurally related to DHPS, is produced through the reduction of dihydroxyacetone phosphate or glycerol phosphorylation. No enzymatic activity was detected when glycerol phosphate was incubated with *Cp*HpsN and NAD^+^. Similarly, no activity was observed when l-histidinol was incubated with *Cp*HpsN and NAD^+^. Based on structural analogy with *R*-SL, we examined whether the 2-amino substituted analogue l-cysteic acid (*R*-cysteic acid) was an inhibitor of *Cp*HpsN. At [*R*-DHPS] = 1.0 mM and [NAD^+^] = 0.3 mM, l-cysteate inhibited *Cp*HpsN with IC_50_ = 2.4 mM (Fig. S5†).

### 2.3. 3D structure of HpsN reveals an octahedral zinc centre, cofactor binding pocket and conformational change upon sulfonate binding

To determine the structural basis of catalysis of *Cp*HpsN we obtained a series of 3D structures. Initially, the protein crystallized as the *Cp*HpsN·Zn^2+^ complex in space group *P*2_1_2_1_2_1_ and the 3D structure was solved and refined to 1.94 Å resolution. Crystals of the *Cp*HpsN·Zn^2+^·NADH complex were obtained by soaking *Cp*HpsN·Zn^2+^ crystals with NADH and diffracted to 2.24 Å. Attempts to soak the *Cp*HpsN·Zn^2+^ crystals with high concentrations of NADH/NAD^+^, and SL or DHPS were unsuccessful to obtain a substrate or product bound complex. We therefore rescreened crystallization conditions for *Cp*HpsN with NADH and product. This was successful and provided crystals of the *Cp*HpsN·Zn^2+^·NADH·*R*-SL complex in a different space group (*P*2_1_) that diffracted to 1.57 Å resolution. *Cp*HpsN structures in both space groups contain a dimer in the asymmetric unit.

**Fig. 4:**
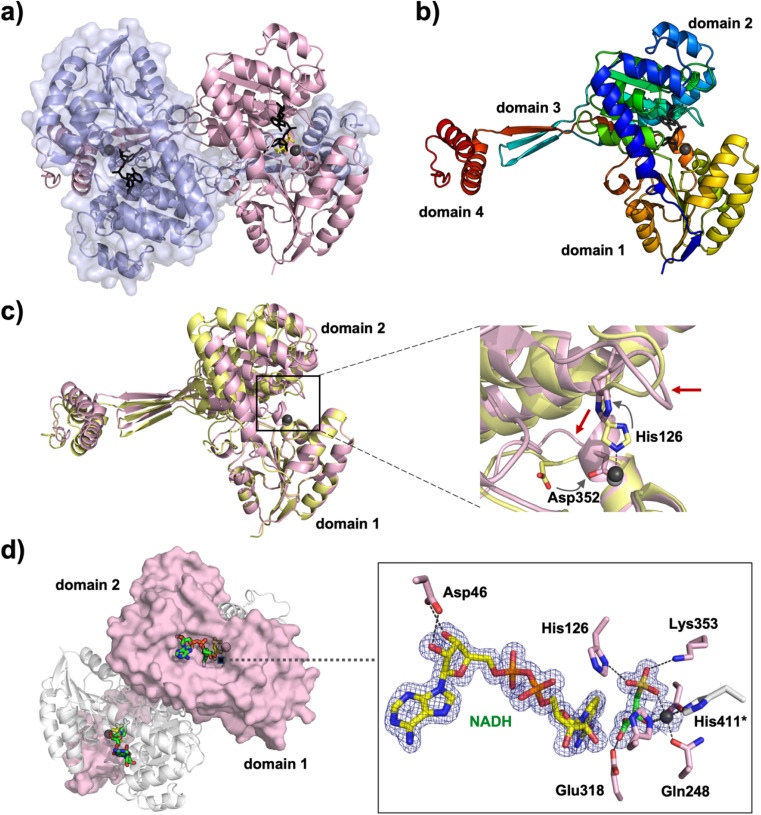
3D crystal structures of *Cp*HpsN. (a) Structure of domain-swapped *Cp*HpsN dimer of the ternary complex with *R*-SL and NADH (*Cp*HpsN·Zn^2+^·NADH·*R*-SL). Chain A in light pink is displayed in cartoon representation while chain B (light slate) in cartoon and surface representations with Zn^2+^ shown as grey spheres. Cofactor, NADH, is depicted in stick representation in black. (b) Monomer of *Cp*HpsN is shown in cartoon representation with rainbow color scheme (N-terminus in blue and C-terminus in red). (c) Superposition of the structures of *Cp*HpsN·Zn^2+^·NADH·*R*-SL (light pink) and *Cp*HpsN·Zn^2+^·NADH (yellow). Domain 1 was used for least squares superposition for comparison of the local structural differences in flexible loops (highlighted with red arrows) near the active site in the zoomed panel. (d) NADH and *R*-SL binding in the active site. Chain A is shown in surface representation (light pink) while chain B in cartoon representation (light grey). Electron density maps shown in blue mesh are 2Fo–Fc maps contoured at 1*σ*.

**Fig. 5:**
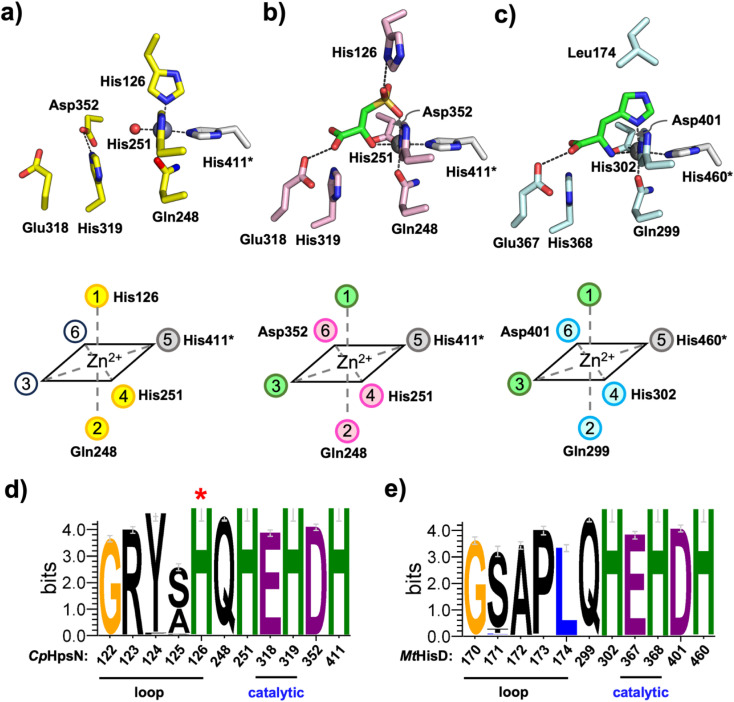
Comparison of Zn-coordinating residues and proposed catalytic residues between *Cp*HpsN and *Mt*HisD. (a)–(c) Zn centres in *Cp*HpsN·Zn^2+^ (a), *Cp*HpsN·Zn^2+^·NADH·*R*-SL (b), and *Mt*HisD·Zn^2+^·NAD^+^·l-histidine (PDB entry 5VLD) (c) with schematic coordination geometry shown below. Carbon atoms in the residues from chain A are colored in light pink (*Cp*HpsN) and light cyan (*Mt*HisD) while those from chain B are in light grey. Carbon atoms in the product (*R*-SL and histidine) are shown in green and Zn^2+^ shown in grey spheres. (d) and (e) Weblogo diagrams (*Cp*HpsN (d) and *Mt*HisD (e)) showing unique sequence motifs identified herein, and unique zinc- and ligand-binding histidine residue (red *) for HpsN.

*Cp*HpsN forms a domain-swapped tight dimer with 28% of surface area buried at the dimer interface, with the interface featuring extensive hydrophobic and hydrogen-bond interactions (Fig. 4a). Each protomer consists of four distinct domains; domains 1 and 2 are globular and contain the active sites while domain 3 is engaged in dimerization (Fig. 4b and S6†). Domain 4 lies at the C-terminus and contributes to the active site and is also involved in dimerization through a metal binding site. Coordination of Zn^2+^ involves three residues from one monomer and one residue (His411*; * denotes the other monomer in the dimer) from the other monomer (Fig. 5 and S7†), showing that the enzyme forms an obligate dimer. Zn^2+^ adopts an octahedral coordination geometry in *Cp*HpsN. While most Zn metalloenzymes feature tetrahedral Zn coordination, with direct participation of Zn^2+^ in catalysis by activating a water molecule, it has been shown that within various plant and bacterial histidinol dehydrogenases, Zn^2+^ adopts an octahedral geometry.^23,24^

Comparison of the 3D structures of the *Cp*HpsN·Zn^2+^ and *Cp*HpsN·Zn^2+^·NADH·*R*-SL complexes reveals that binding of NADH and *R*-SL causes a significant movement and closure of domains 1 and 2 (Fig. 4c and S8a†). The closure of the two domains results in a decrease in the distance from the tip (Arg153) of domain 1 to the tip (Thr284) of domain 2 from 21.5 Å to 13.5 Å (Fig. S9†). Analysis of the interfaces using the Protein Interfaces, Surfaces and Assemblies interactive tool^25^ indicates that the *Cp*HpsN·Zn^2+^·NADH·*R*-SL complex forms a more compact dimer *versus Cp*HpsN·Zn^2+^, with the increased buried surface area from 9620 to 13 250 Å^2^ and decrease in Δ*G*^diss^ from 84.1 to 98.7 kcal mol^−1^. The root-mean square deviation of the *Cp*HpsN·Zn^2+^ and *Cp*HpsN·Zn^2+^·NADH·*R*-SL structures is 2.0 Å over 407 common Cα positions. A further difference in the two structures is a change in positions of His126 and Asp352, as well as in the flexible loops in which these residues are located (Fig. 4c). This will be discussed in more detail below.

A structure-based search using Foldseek^26^ with the ‘open’ *Cp*HpsN·Zn^2+^ structure as query identified *E. coli* histidinol dehydrogenase (*Ec*HisD, PDB 1KAR),^23^ in complex with Zn^2+^ and histamine (a substrate/product analogue), as the closest structural homologue (sequence identity 29.7%, *E*-value = 2.6 × 10^−32^) (Fig. S8b†). However, using the ‘closed’ *Cp*HpsN·Zn^2+^·NADH·*R*-SL structure as query, the search yielded as the top ranked target *Medicago truncatula* histidinol dehydrogenase (*Mt*HisD, PDB 5VLD),^24^ in complex with Zn^2+^, NAD^+^ and l-histidine (sequence identity of 33.6%; *E*-value = 4.5 × 10^−38^) (Fig. S8c†). Thus, when cofactor and product are bound *Cp*HpsN adopts the same ‘closed’ conformation as *Mt*HisD, while when crystals were grown without ligands, the resulting crystal structure adopts the same ‘open’ conformation as *Ec*HisD.

### 2.4. 3D structure of *Cp*HpsN·Zn^2+^·NADH·*R*-SL complex

The crystal structure of the *Cp*HpsN·Zn^2+^·NADH·*R*-SL complex reveals NADH and *R*-SL bound with full occupancy in the active site, in close proximity and aligned for hydride transfer (Fig. 4d and S9b†). The nucleotide moiety of NADH is bound to domain 2 and extends towards the Zn center in domain 1. The adenine ring stacks between two hydrophobic residues, Leu45 and Phe205. Asp46 makes hydrogen bond interactions with the 2′- and 3′-OH groups and there is insufficient space to accommodate the phosphate group in NADP^+^, which explains the preference of NAD^+^*versus* NADP^+^. The nicotinamide group is placed almost parallel to the carboxylate group of *R*-SL with the C4 of the nicotinamide group 3.1 Å from the carboxyl carbon of *R*-SL. The carboxylate group of *R*-SL is engaged in hydrogen bond interactions with the carbonyl oxygen of His359, Ser 226, and Glu318. The secondary hydroxyl group and sulfonate group of *R*-SL are coordinated to the Zn center. The sulfonate group of *R*-SL is further supported by hydrogen bond interactions with His126, Lys353 and a water molecule.

In contrast, the crystal structure of *Cp*HpsN·Zn^2+^·NAD^+^, obtained by soaking crystals of *Cp*HpsN·Zn^2+^ in the open conformation with NAD^+^, represents a nonproductive complex where the electron density for the nicotinamide group is disordered in the crystal structure and therefore, modelled with zero occupancy (Fig. S9a†). The distance between the projected position of the nicotinamide and the Zn center is beyond the expected distance for a hydride transfer from the substrate. Therefore, the closure of the domains 1 and 2 are essential for productive complex formation.

In addition to *Cp*HpsN·Zn^2+^·NADH·*R*-SL complex, we also obtained crystals of the *Cp*HpsN·Zn^2+^·NADH·l-cysteate complex that diffracted to 1.75 Å using a similar co-crystallization approach (Fig. S10c†). The overall structure and the active site coordination of the cysteate complex is essentially identical to that of the *Cp*HpsN·Zn^2+^·NADH·*R*-SL structure, demonstrating that this inhibitor functions through mimicry of *R*-SL.

### 2.5. Changes in the zinc coordination sphere of *Cp*HpsN upon binding NADH and *R*-SL

Comparison of the *Cp*HpsN·Zn^2+^ and *Cp*HpsN·Zn^2+^·NADH·*R*-SL structures reveals rearrangement in the octahedral coordination environment at the zinc center. In the *Cp*HpsN·Zn^2+^ structure, Zn^2+^ is coordinated by four amino acid residues: three histidine residues (His126, His251 and His411*) and Gln248, and the remaining two equatorial sites (#3 and #6) were modelled as water (Fig. 5a and S7a†). In the *Cp*HpsN·Zn^2+^·NADH·*R*-SL structure, a conformational change occurs in the connecting loops comprised of residues 120–128 and 348–354 (Fig. 4c) leading to a rearrangement about zinc involving the axial-coordinating His126 (site #1) changing to an equatorial-coordinating Asp352 (site #6) (Fig. 5b and S7b†). In the Ramachandran plot of the *Cp*HpsN·Zn^2+^ structure, Asp352 is placed in the right-handed α helix region, while in the *Cp*HpsN·Zn^2+^·NADH·*R*-SL structure this residue lies in the β-sheet region, demonstrating a significant difference in the loop conformation near the active site. In a similar manner, Ala121 in the connecting loop (residues 120–128), which is placed in the left-handed α-helix region in the *Cp*HpsN·Zn^2+^ structure, moves to the β-sheet region in the *Cp*HpsN·Zn^2+^·NADH·*R*-SL structure. These changes about the Zn center in the *Cp*HpsN·Zn^2+^·NADH·*R*-SL structure accommodates binding of *R*-SL, with the sulfonate group occupying axial site #1 and the secondary hydroxyl occupying equatorial site #3. The conformational change of the connecting loops (residues 120–128 and 348–354) in the *Cp*HpsN·Zn^2+^·NADH·*R*-SL structure places His126 away from the Zn center so that it no longer coordinates to the metal, but now interacts with the sulfonate group along with Lys353 (Fig. 4d and 5b).

### 2.6. Comparison of zinc coordination spheres in DHPS and histidinol dehydrogenases

The binding mode observed for the product, *R*-SL, bound to *Cp*HpsN is strikingly similar to that observed for product, l-histidine, bound to *Mt*HisD,^24^ and for substrate, l-histidinol, bound to *Ec*HisD,^23^ in the respective complexes that also contain Zn^2+^ and nicotinamide cofactors (Fig. 5 and S10†). All three structures contain an octahedral zinc center with two equatorial His residues (with one drawn from the second chain in the dimer), an equatorial Asp residue, and an axial Gln residue. The substrate/product is bound with the amino/hydroxyl group at equatorial site #3, while the sulfonate or imidazole groups occupy the remaining axial site #1.

A notable difference between the HpsN and HisD structures is the absence of an equivalent histidine residue to His126 of *Cp*HpsN in HisD. This histidine residue is conserved within HpsN homologues, but not within HisD homologues, where it is predominantly Leu. Therefore, the conformational changes about the zinc center are not observed in HisD structures. Multiple sequence alignments reveals an HpsN-specific motif that distinguishes homologues of HisD: G-S-A-P-L, from homologues of HpsN: G-R-Y-A/S–H. The final His in the HpsN-specific motif (His126 in *Cp*HpsN) is the axial Zn^2+^-coordinating residue observed in the *Cp*HpsN complex lacking *R*-SL that instead binds the sulfonate in the *Cp*HpsN·Zn^2+^·NADH·*R*-SL structure (Fig. 5d and e). Possibly, this residue may assist in maintaining a high affinity for Zn^2+^ in the absence of substrate/product, and for discrimination of *R*-SL *versus*l-histidinol.

### 2.7. Site-directed mutagenesis supports roles for Glu318, His319 and Asp352 in catalysis and metal coordination

A two-phase mechanism for *Ec*HisD has been proposed on the basis of kinetic analysis, site-directed mutagenesis, and structural studies.^23,24^ In the first phase l-histidinol binds to *Ec*HisD with the amino and imidazole groups coordinated to sites #1 and #3. His327 acts as general base to deprotonate the hydroxyl group of l-histidinol and promote hydride transfer to NAD^+^ to generate l-histidinal. Next, Glu326 acts as a general base to promote nucleophilic addition of water to the intermediate aldehyde while protonated His327 acts as general acid, generating l-histidinal hydrate. In the second phase, His327 again acts as general base to deprotonate the aldehyde hydrate and promote a second hydride transfer to a second molecule of NAD^+^, forming l-histidine. Glu326 and His327 in *Ec*HisD are conserved in *Cp*HpsN (Glu318 and His319), occupy approximately similar positions, and could conceivably play the same roles.

To probe the roles of Glu318, His319 and Asp352, we conducted site-directed mutagenesis to convert each residue independently to Ala. The E318A and H319A mutants of *Cp*HpsN suffered 580-fold and 240-fold reductions in (*k*_cat_/*K*_M_)^app^ values, respectively, which was mainly due to a reduction in *k*^app^_cat_ values (Fig. S11† and Table 2). No activity was detected for the D352A mutant of *Cp*HpsN, most likely due to the inability to form a productive zinc-coordination complex with substrate. The 3D structure of the *Cp*HpsN D352A mutant in complex with Zn^2+^ and NAD^+^ was solved and refined to 2.23 Å resolution and is essentially identical to the *Cp*HpsN·Zn^2+^·NADH structure in the ‘open’ conformation (Fig. S12a–c†). In contrast, the structure of *Cp*HpsN H319A in complex with *R*-DHPS and NADH (refined to 2.01 Å resolution) adopts the closed conformation and is similar to the *Cp*HpsN·Zn^2+^·NADH·*R*-SL structure in that the sulfonate and secondary hydroxyl groups participate in the octahedral zinc coordination (Fig. S12d and e†). The 3D structure of the ‘closed’ *Cp*HpsN H319A·Zn^2+^·NADH·*R*-DHPS complex displays a productive geometry with the primary hydroxyl carbon of DHPS positioned 3.3 Å away from C4 of the nicotinamide group. In this complex, the Zn and *R*-DHPS sites are not fully occupied, and the final coordinates are modelled with the occupancy of 0.7, whereas the cofactor NADH has a full occupancy. The partial occupancy of zinc presumably arises due to incomplete reconstitution during purification. However, the concentration of *R*-DHPS used during crystallization was in excess (>100×) and is not a limiting factor. This observation therefore suggests that zinc is essential for ligand binding in the active site. Collectively, these data provide evidence for a role for Asp352 in zinc coordination to form a catalytically productive complex, and for Glu318 and His319 in the catalytic mechanism of *Cp*HpsN.

**Table 2:** Kinetic analysis of *Cupriavidus pinatubonensis* DHPS-3-dehydrogenase HpsN mutants^a^

Mutation	Variable substrate	Constant substrate	Concentration (mM)	*k* ^app^_cat_ (s^−1^)	*K* ^app^_M_ (mM)	(*k*_cat_/*K*_M_)^app^ (mM^−1^ s^−1^)
E318A	*R*-DHPS	NAD^+^	0.30	5.1 × 10^−3^ ± 0.01	2.8 ± 0.3	1.8 × 10^−3^ ± 0.03
H319A	*R*-DHPS	NAD^+^	0.30	3.7 × 10^−3^ ± 0.03	1.2 ± 0.3	3.1 × 10^−3^ ± 0.10
D352A	*R*-DHPS	NAD^+^	0.30	ND	ND	ND

a ND, no activity detected.

### 2.8. Taxonomic distribution of HpsN across different pathways of DHPS catabolism

DHPS-3-dehydrogenases are involved in three distinct pathways that we classify based on their utilization of different sulfolyases: Fe^2+^-dependent SuyAB lyase, ThDP-dependent Xsc sulfoacetaldehyde acetyltransferase, or PLP-dependent CuyA cysteate lyase (Fig. 2). To investigate the taxonomic distribution and pathway occurrence of HpsN homologues, we employed sequence similarity networks (SSN).^27^ By conducting a BLAST search of the UniProt database using *C. pinatubonensis* HpsN sequence as a query, we retrieved 1000 sequences (belonging to PFAM protein family PF00815) for a wide range of bacteria. Subsequently, we used these sequences to retrieve the genome neighborhood diagrams within a ±10 open reading frame window of the *hpsN* genes using the Enzyme Function Initiative web tools (https://efi.igb.illinois.edu/efi-gnt/).^28^ For further analysis, we selected 272 HpsN sequences that met the criteria that they had *hpsO* (from family PF13561) and *hpsP* (from family PF00107-PF08240) genes located in their proximity.

**Fig. 6:**
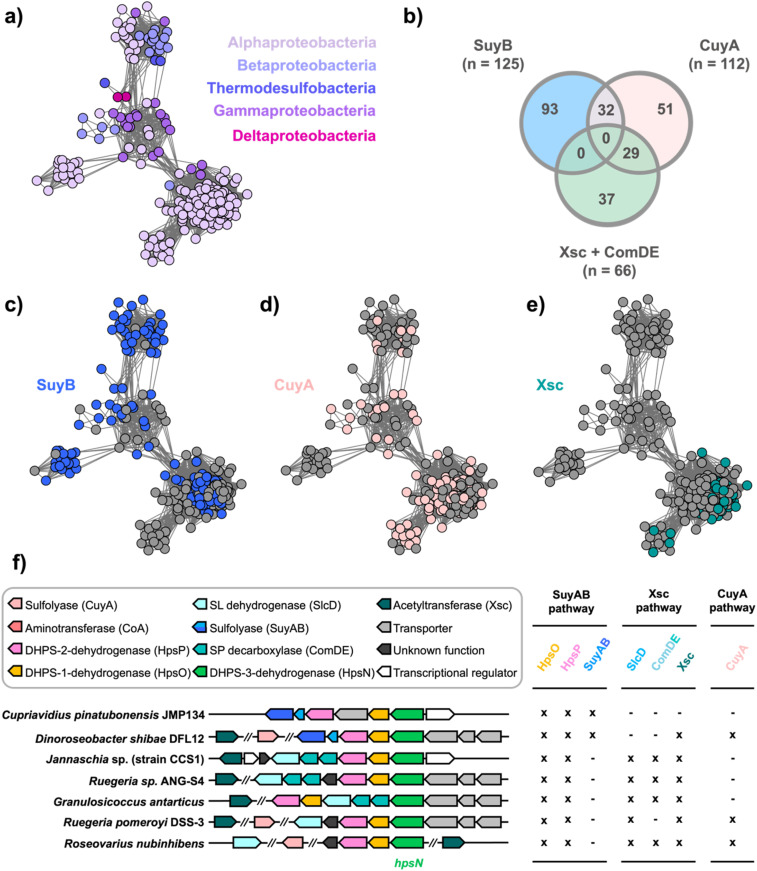
Sequence similarity network (SSN) of HpsN homologues and representative organisms encoding HpsN-dependent SL biomineralization pathways. (a) SSN at alignment score = 170 (*i.e.*, > 62.9% identity) colored based on taxonomy of organisms harboring *hpsN* gene. (b) Venn diagram showing occurrence of SL biomineralization pathways in organisms harboring *hpsN* genes based on presence of genes encoding indicated SL modifying proteins. (c) HpsN SSN colored based on co-occurrence of *hpsN* and *suyB* genes. (d) HpsN SSN colored based on co-occurrence of *hpsN* and *cuyA* genes. (e) HpsN SSN colored based on co-occurrence of *hpsN* and *xsc* genes. (f) Bacterial gene diagrams encoding representative short-chain organosulfonate biomineralization pathways containing the *hpsN* gene, and assignment of proteins into various SL biomineralization pathways.

The 272 HpsN sequences were used to generate a SSN at varying alignment scores (AS) (Fig. S13†). An SSN with AS = 170 was chosen as it generated an SSN with a single cluster, but which naturally segregated into interconnected sub-clusters. These sub-clusters exhibited high intra-subcluster connectivity and low inter-subcluster connectivity, and their fine structure aligned with taxonomy at the class level (Fig. 6a). HpsN sequences were distributed across a range of bacterial classes including Alphaproteobacteria, Betaproteobacteria, Gammaproteobacteria, Deltaproteobacteria and Desulfobacteria.

In Fig. 6 we provide examples of gene clusters from organisms that encode the three different pathways. *Cupriavidus pinatubonensis* JMP134 and *Dinoroseobacter shibae* DFL12 represent the SuyAB pathway, and contain genes encoding HpsNOP for epimerization of DHPS and oxidation to SL; as well as SuyAB for cleavage of C–S bond of SL to give pyruvate. *Jannaschia* sp. (strain CCS1), *Ruegeria* sp. ANG-S4, and *Granulosicoccus antarticus* are representatives of the Xsc pathway and include genes encoding HpsNOP for epimerization of DHPS and oxidation to SL; SlcD for oxidation of SL to sulfopyruvate; ComDE for decarboxylation of sulfopyruvate to sulfoacetaldehyde; and Xsc for cleavage of the carbon sulfur bond to give acetyl phosphate and sulfite. *Ruegeria pomeroyi* DSS-3 and *Roseovarius nubinhibens* are representatives of the CuyA pathway and feature genes encoding HpsNOP for epimerization of DHPS and oxidation to SL; SlcD for oxidation of SL to sulfopyruvate; and CuyA, which catalyzes deamination, sulfite elimination and formation of pyruvate. The identity of the genes encoding aminotransferase (CoA) that converts sulfopyruvate to cysteate, and the cysteate racemase (CuyB) in *R. pomeroyi*, are unknown.^19,20^

To further study the neighboring genes, we employed the EFI-GNT tools to examine open reading frames within a ±10 range. The genes were used to construct an SSN of neighbors (SSNN). The SSNN revealed isofunctional clusters for the three sulfolyases: Xsc (*n* = 12), SuyB (*n* = 58; including four members with a fused SuyA-SuyB protein), and CuyA (*n* = 34). However, the total number of members of these three clusters (*n* = 104) was much smaller than the 272 HpsN sequences used in the original SSN. It is worth noting that sulfolyases are not always co-located with HpsNOP, leading us to manually conduct a BLASTp search for each pathway protein in the organisms that lacked adjacent genes encoding sulfolyase enzymes. This search revealed that 125 bacteria contained SuyB, 112 contain CuyA, and 66 contain both Xsc and ComDE.

Nodes in the HpsN SSN were colored based on the presence of the *hpsN* gene in a bacterium containing putative pathways for the breakdown of SL through different sulfolyases: the SuyAB pathway (93/272; containing *hpsNOP*, *suyB*); the CuyA pathway (51/272; containing *hpsNOP*, *cuyA*); and the Xsc pathway (37/272; containing *hpsNOP*, *comDE*, *xsc*) (Fig. 6c–e). To categorize whether individual organisms contained multiple pathways we employed a Venn diagram (Fig. 6b). This enumerates organisms containing both the SuyAB and CuyA pathways (*n* = 32), and organisms containing both the Xsc and CuyA pathways (*n* = 29). The SuyAB pathway was found in all the Proteobacteria and Thermodesulfobacteria candidates, the CuyA pathway was found in some of the Alphaproteobacteria, Gammaproteobacteria and Thermodesulfobacteria; while the Xsc pathway was only found in the Alphaproteobacteria.

## 3 Discussion

DHPS 3-dehydrogenases catalyze the oxidation of DHPS to SL. In this study, using the individual enantiomers of DHPS, we show that HpsN from *C. pinatubonensis* (*Cp*HpsN) exhibits a 10^6^-fold selectivity for *R*-DHPS and a strict dependence on NAD^+^. The oxidation of *R*-DHPS to *R*-SL requires 2 equivalents of NAD^+^ for a 4-electron oxidation and is expected to proceed *via* the intermediate *R*-SLA. Despite HPLC analysis of incomplete reactions not allowing detection of SLA (data not shown), kinetic analysis revealed that SLA, presumably of the *R* configuration, is superior to *R*-DHPS as a substrate in terms of (*k*_cat_/*K*_M_)^app^ values. HpsN displays specificity for the sulfonated substrate *R*-DHPS and does not exhibit detectable activity on the structurally related phosphate analogue, glycerol-1-phosphate or l-histidinol. DHPS-3-dehydrogenases share sequence similarities with histidinol dehydrogenases, which catalyze the 4-electron oxidation of l-histidinol to l-histidine and are also NAD^+^-dependent enzymes. In a similar manner, histidinol dehydrogenases also accept the intermediate histidinal as a substrate.^29^

Histidinol dehydrogenases from *E. coli* and other organisms are Zn^2+^ dependent enzymes. Therefore, *Cp*HpsN reconstituted with Zn^2+^ was used for all kinetic and structural studies. Treatment of Zn^2+^-loaded *Cp*HpsN with EDTA resulted in a loss of activity, confirming that *Cp*HpsN is a metallo-enzyme. A metal screen of a range of divalent metals identified good activity for a wide range of transition and main group dications, with Zn^2+^ among the most active. The 3D structure of *Cp*HpsN reveals a dimer with similar fold and quaternary structure to *E. coli* and *M. truncatula* histidinol dehydrogenases. All enzymes contain an octahedral metal binding site, formed by amino acid residues from both protomers within the dimer, and binds substrate in similar orientations about the zinc centre, but with the sulfonate group of *R*-SL taking the place of the imidazole group of l-histidine.

Comparison of complexes of *Cp*HpsN·Zn^2+^ with *Cp*HpsN·Zn^2+^·NADH·*R*-SL reveals conformational changes in two flexible loops that allow remodeling of the coordination environment about the zinc center. In the absence of *R*-SL, the axial site is occupied by a conserved histidine residue (His126) in one loop, which is displaced in the presence of *R*-SL, while a conserved aspartate (Asp352) in another loop is repositioned and occupies an axial site. This remodeling of the zinc coordination environment appears to be unique to DHPS 3-dehydrogenases, as histidinol dehydrogenases lack a residue equivalent to His126, and identical coordination environments being observed with, and without, l-histidinol or l-histidine bound.

**Fig. 7:**
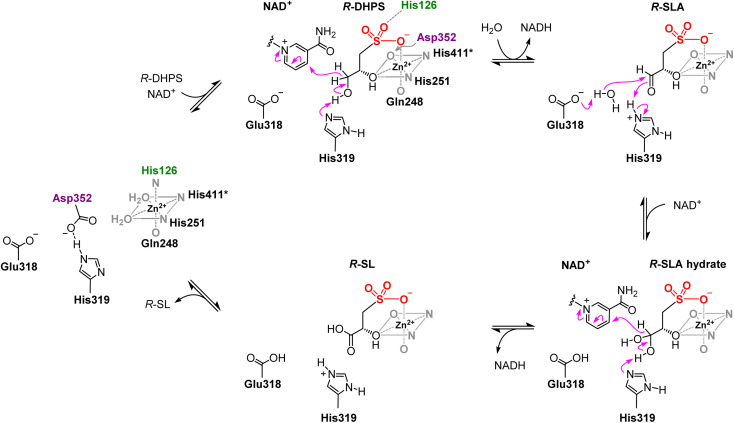
Proposed mechanism of *Cp*HpsN involving oxidation of *R*-DHPS to *R*-SL *via R*-SLA.

Our data collectively reveal a characteristic sequence motif that distinguishes DHPS and histidinol dehydrogenases (Fig. 5d and e). For *Cp*HpsN this motif comprises residues 122–126, with the final His126 being conserved among HpsN homologues and binding at the axial site of Zn^2+^ in the absence of substrate and cofactor. This residue relinquishes its role in zinc coordination to Asp352 upon substrate and cofactor binding, and instead participates in substrate recognition and coordination through a hydrogen bond with the sulfonate group of *R*-DHPS. In the case of *Mt*HisD, the equivalent motif comprises residues 170–174 and lacks the terminal His residue found in HpsN homologues. Kinetic analysis of the individual *Cp*HpsN Glu318Ala, His319Ala, Asp352Ala variants showed large decreases in catalytic activity, consistent with these residues playing a role in zinc coordination and the catalytic mechanism of *Cp*HpsN. By analogy with the two-phase mechanism proposed for *Ec*HisD,^23,24^ we propose that catalysis by *Cp*HpsN involves initial binding of *R*-DHPS to one axial and one equatorial sites of Zn (sites #1 and #3) (Fig. 5b). We propose that, like *Ec*HisD, His319 acts as a general base to deprotonate the primary hydroxyl of *R*-DHPS and promote hydride transfer to NAD^+^ to generate *R*-SLA (Fig. 7). Next, Glu318 acts as general base, promoting nucleophilic addition of water to *R*-SLA, while protonated His319 acts as general acid, generating *R*-SLA hydrate. In the second phase, His319 acts as general base to deprotonate the *R*-SLA hydrate and promote a second hydride transfer to a second molecule of NAD^+^, forming *R*-SL.

## 4 Conclusions

DHPS-3-dehydrogenases (HpsN) are key enzymes that facilitate DHPS degradation through oxidation to SL, providing entry to multiple routes for its complete catabolism involving sulfolyases SuyAB, CuyA and Xsc. In this work we show that *Cp*HpsN (and, by extension, other HpsN homologues) oxidizes *R*-DHPS to give *R*-SL. This stereochemical preference is opposite to that of SLA dehydrogenase (GabD), which is linked to the output of sulfoglycolysis, and oxidizes *S*-SLA to *S*-SL.^15^ It has been proposed that *R*-DHPS can be formed from *S*-DHPS through the action of the oxidoreductases HpsO–HpsP, but it is unknown whether there are other natural pathways that directly produce *R*-DHPS. The significance of HpsN in marine DHPS metabolism is underscored by the measurement of bacterial *hpsN* transcripts of 1.0 × 10^6^ to 2.5 × 10^7^ l^−1^ along a 230 km coastal-to-open ocean transect in the eastern North Pacific during an algal bloom.^8^ This work illuminates the molecular mechanism and stereochemical preference of HpsN, enhancing our understanding of this key enzyme in the biogeochemical sulfur cycle.

## Data availability

The ESI† includes experimental and additional details on protein biochemistry (Fig. S1†), enzyme kinetics (Fig. S2–S5 and S11†), 3D structural data (Fig. S6–S10 and S12†), bioinformatics (Fig. S13 and S14†), and structural statistics (Table S1†). A separate file contains accession codes and associated data for proteins used in bioinformatics analysis. Structural data (atomic coordinates) have been deposited with the Protein Data Bank (PDB accession codes: 8v35, 8v36, 8v37, 9CP7, 9CP8, and 9CP9).

## Author contributions

S. J. W. conceived the project. M. L. performed molecular biology, protein expression and structural biology. L. B. and A. N. performed enzyme kinetics. A. K. conducted bioinformatics. M. L, L. B., A. K. and S. J. W. designed experiments, analyzed data and wrote the manuscript.

## Conflicts of interest

The authors declare no competing interests.

## Supplementary Material

SC-OLF-D4SC05114A-s001

SC-OLF-D4SC05114A-s002
