# Supplementary material for: Structural and kinetic insights into the stereospecific oxidation of R-2,3-dihydroxypropanesulfonate by DHPS-3-dehydrogenase from Cupriavidus pinatubonensis
Source: Chem Sci. 2024 Sep 9;15(38):15757–68. doi: 10.1039/d4sc05114a (PMC11382572; doi:10.1039/d4sc05114a)
Supplement: SC-015-D4SC05114A-s002 [file SC-015-D4SC05114A-s002.pdf]

Supplementary Information (SI) for Chemical Science.
This journal is © The Royal Society of Chemistry 2024

SUPPLEMENTARY INFORMATION

Structural and kinetic insights into the stereospecific oxidation of *R*-2,3-dihydroxypropanesulfonate by DHPS-3-dehydrogenase from *Cupriavidus pinatubonensis*

Laura Burchill,[a] Arashdeep Kaur,[a] Artur Nastasovici,[a] Mihwa Lee,[a]* Spencer J. Williams[a]*

[a] School of Chemistry and Bio21 Molecular Science and Biotechnology Institute, University of Melbourne, Parkville, Victoria 3010, Australia

* Corresponding authors
Mihwa Lee: mihwa.lee@unimelb.edu.au
Spencer J. Williams: sjwill@unimelb.edu.au

Supplementary Figures

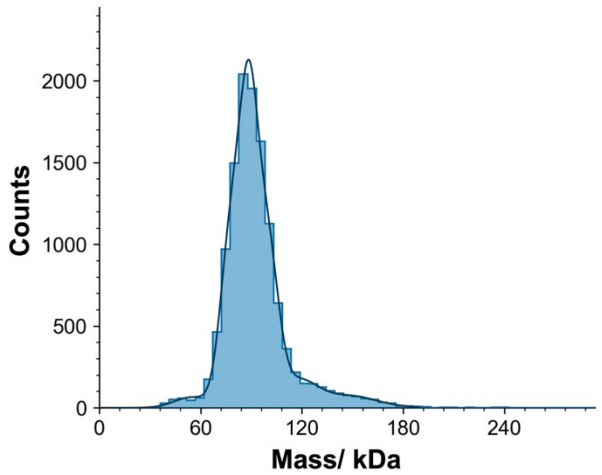

Figure S1. Mass photometry analysis of *Cp*HpsN. A solution of *Cp*HpsN at 50 nM displayed a single peak at an approximate mass of 90 kDa. The calculated dimer mass is 93.8 kDa.

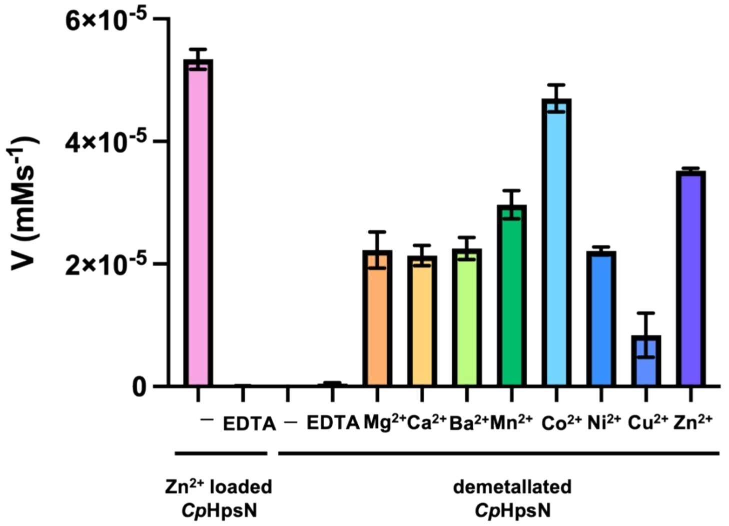

Figure S2. Metal dependence of *Cp*HpsN. Reactions contained [*R*-DHPS] = 1.0 mM and [NAD^+] = 0.3 mM, in 100 mM Bis-Tris Propane buffer, [NaCl] = 200 mM at 20 °C. Divalent metals were present at a concentration of 50 µM and [EDTA] = 50 µM. Reactions were initiated by addition of Zn^{2+} loaded *Cp*HpsN or demetallated *Cp*HpsN. Reactions were performed in triplicate and error bars show SEM.

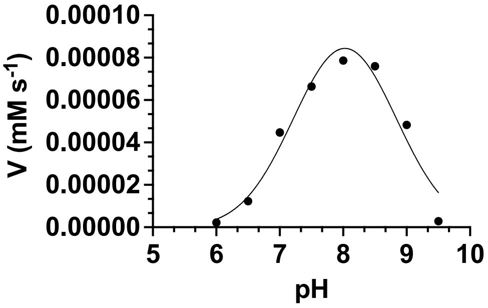

Figure S3. pH dependence of reaction rate of *R*-DHPS oxidation catalyzed by *Cp*HpsN. Reactions contained 100 mM Bis-Tris Propane buffer (at varying pH), [NaCl] = 200 mM, [*R*-DHPS] = 0.2 mM, [NAD^+] = 0.3 mM, and were initiated by addition of [*Cp*HpsN] = 1.67 μM.

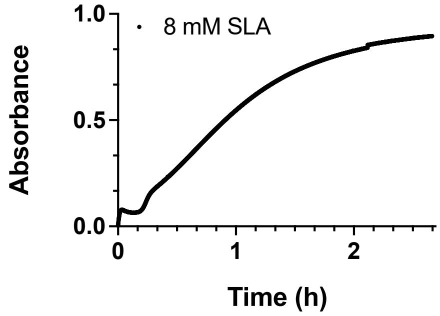

Figure S4. Reaction profile for *Cp*HpsN catalyzed oxidation of racemic SLA. Representative progress curve for *Cp*HpsN catalyzed oxidation of racemic SLA, monitoring formation of NADH (340 nm). Reactions contained [NAD^+] = 0.3 mM, and [SLA] = 8.0 mM. Reactions were performed in triplicate. The maximum absorbances (A_{340}) = 0.955, 0.828, 0.902 for racemic SLA, was observed corresponding to oxidation of 48 ± 1% racemic SLA.

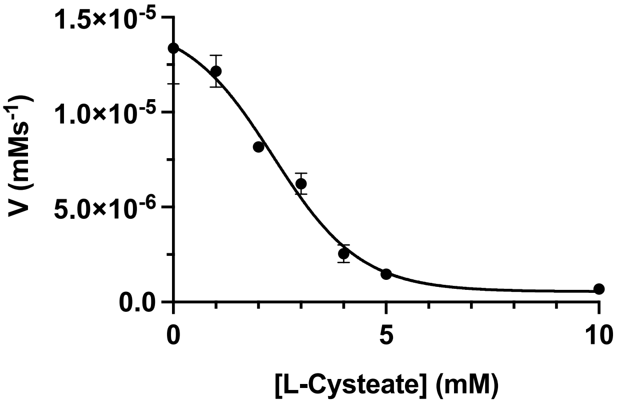

Figure S5. IC_{50} Inhibition Data of L-cysteate against *Cp*HpsN Activity. Reaction of [*R*-DHPS] = 1.0 mM with [NAD^+] = 0.3 mM, conducted in 100 mM Bis-Tris Propane buffer, [NaCl] = 200 mM, [$ZnCl_2$] = 50 μM at 20 °C. Reactions were initiated by *Cp*HpsN at 67.2 nM and performed in triplicate and error bars show SEM. The IC_{50} for L-cysteate was calculated to be 2.4 ± 0.7 mM.

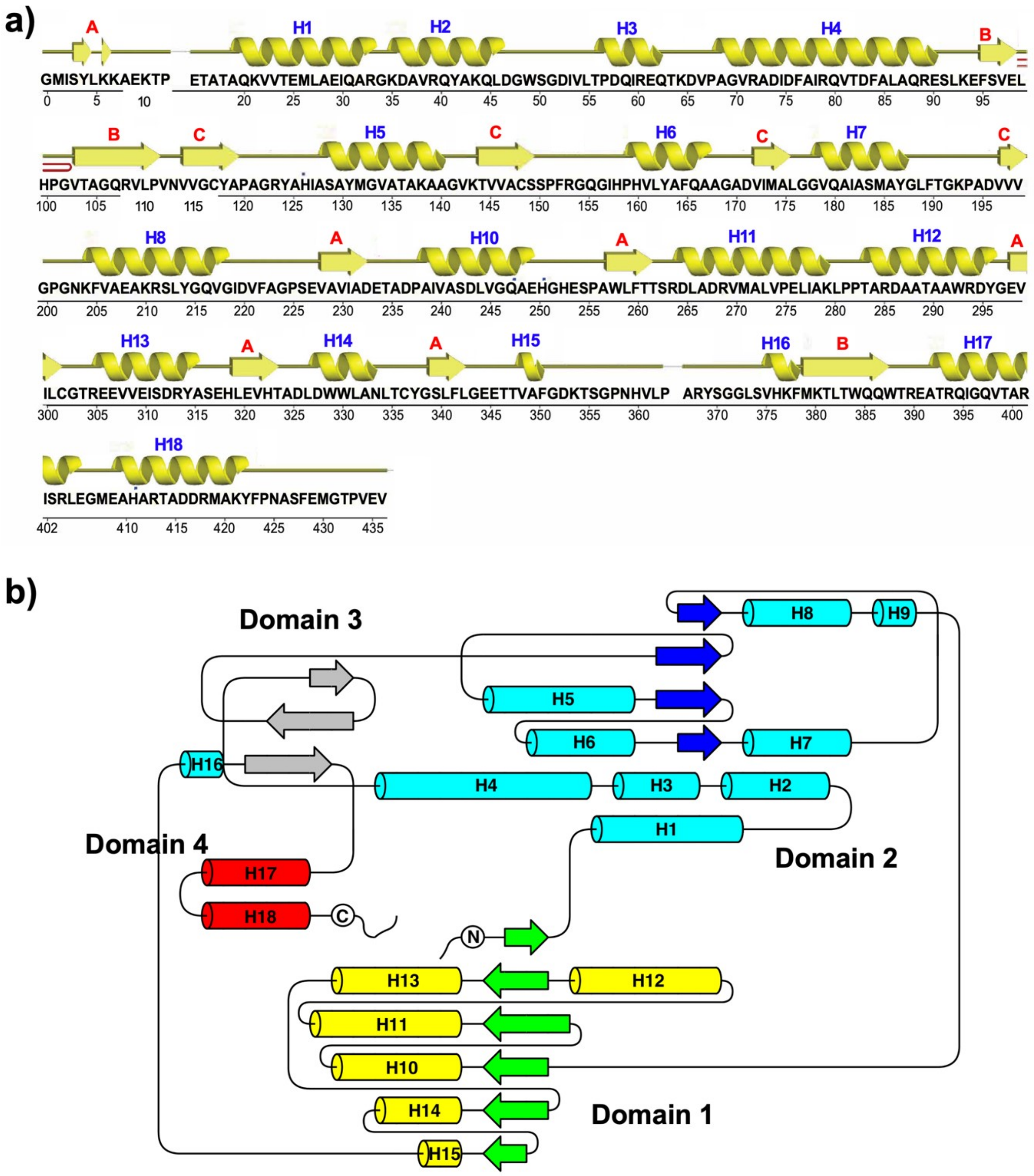

Figure S6. Secondary structure assignment and protein topology of *Cp*HpsN. a) Secondary structure assignment of *Cp*HpsN. α-Helices are labelled H1–H18 while β-strands by their β-sheets, A, B, and C. Image generated using PDBsum.[1]. **b**) Topology diagram of *Cp*HpsN. α-Helices are colored in yellow (domain 1), cyan (domain 2), and red (domain 4), while β-strands in green (domain 1; β-sheet A), blue (domain 2; β-sheet C), and grey (domain 3; β-sheet B), respectively. Image generated using TopDraw.[2]

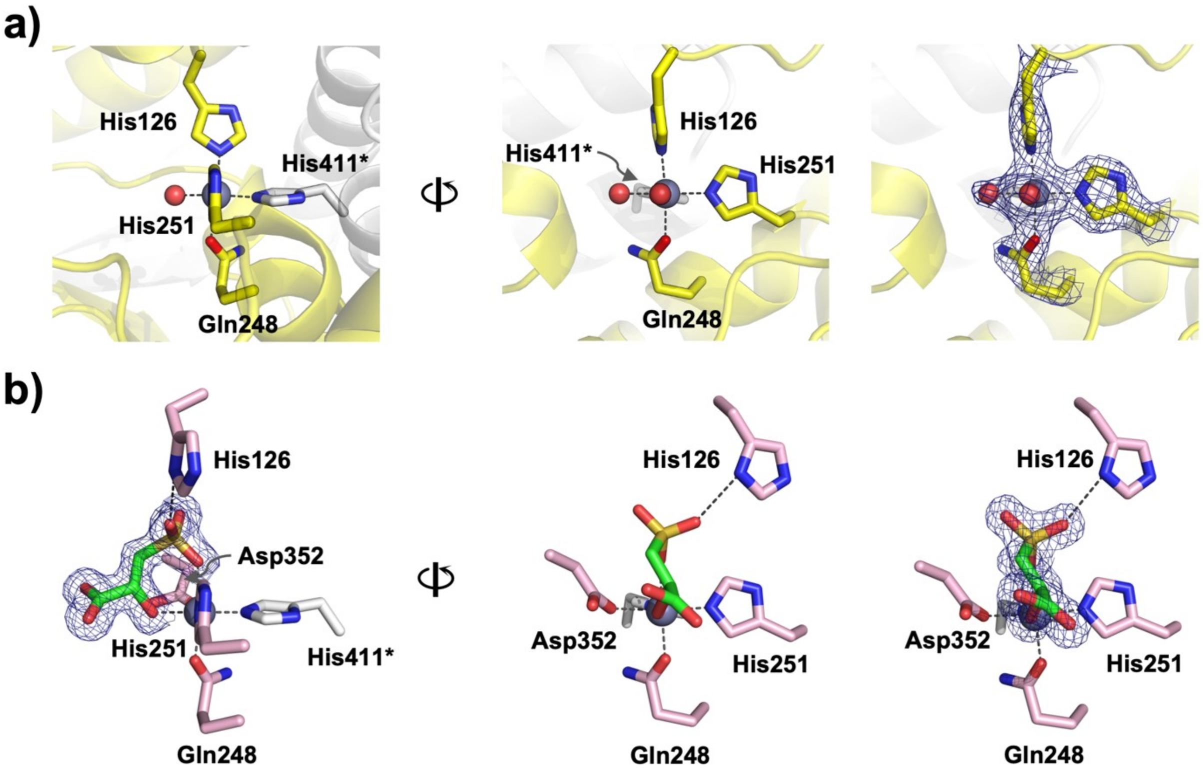

Figure S7. Comparison of Zn^{2+} centres in the crystal structures of apo- and ternary complex of Zn^{2+}-loaded *Cp*HpsN. **a**) Octahedral coordination of Zn^{2+} in the apo-*Cp*HpsN involves three residues from chain A (His126, His251, and Gln248) and one from chain B (His411). The final coordinates deposited in PDB include two water molecules for sites #3 and #6. **b**) Octahedral coordination of Zn^{2+} in the *Cp*HpsN•*R*-SL•NADH ternary complex engages Asp352 in site #6, while *R*-SL occupies sites #1 and 3 as shown in the schematic diagram (**Fig 5**). Electron density maps shown in blue mesh are $2F_o - F_c$ maps contoured at 1 σ.

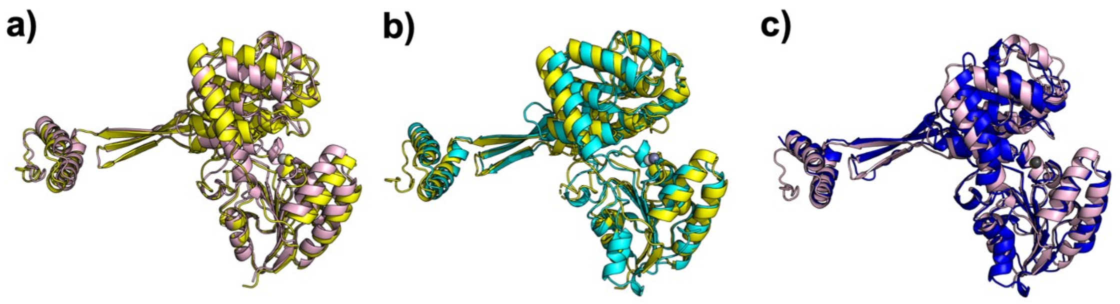

Figure S8. Superposition of *Cp*HpsN with known histidinol dehydrogenase structures by secondar-structure matching (SSM). **a**) *Cp*HpsN•Zn^{2+} structure (yellow. 'open' complex) with *Cp*HpsN•Zn^{2+}•NADH•*R*-SL structure (light pink, 'closed' complex). **b**) Open complexes: *Cp*HpsN•Zn^{2+} structure (yellow) with *Ec*HisD•Zn^{2+}•histamine (cyan, PDB entry 1KAR). **c**) Closed complexes: *Cp*HpsN•Zn^{2+}•NADH•*R*-SL structure (light pink) with *Mt*HisD•Zn^{2+}•NAD^{+}•histidine (blue, PDB entry 5VLD).

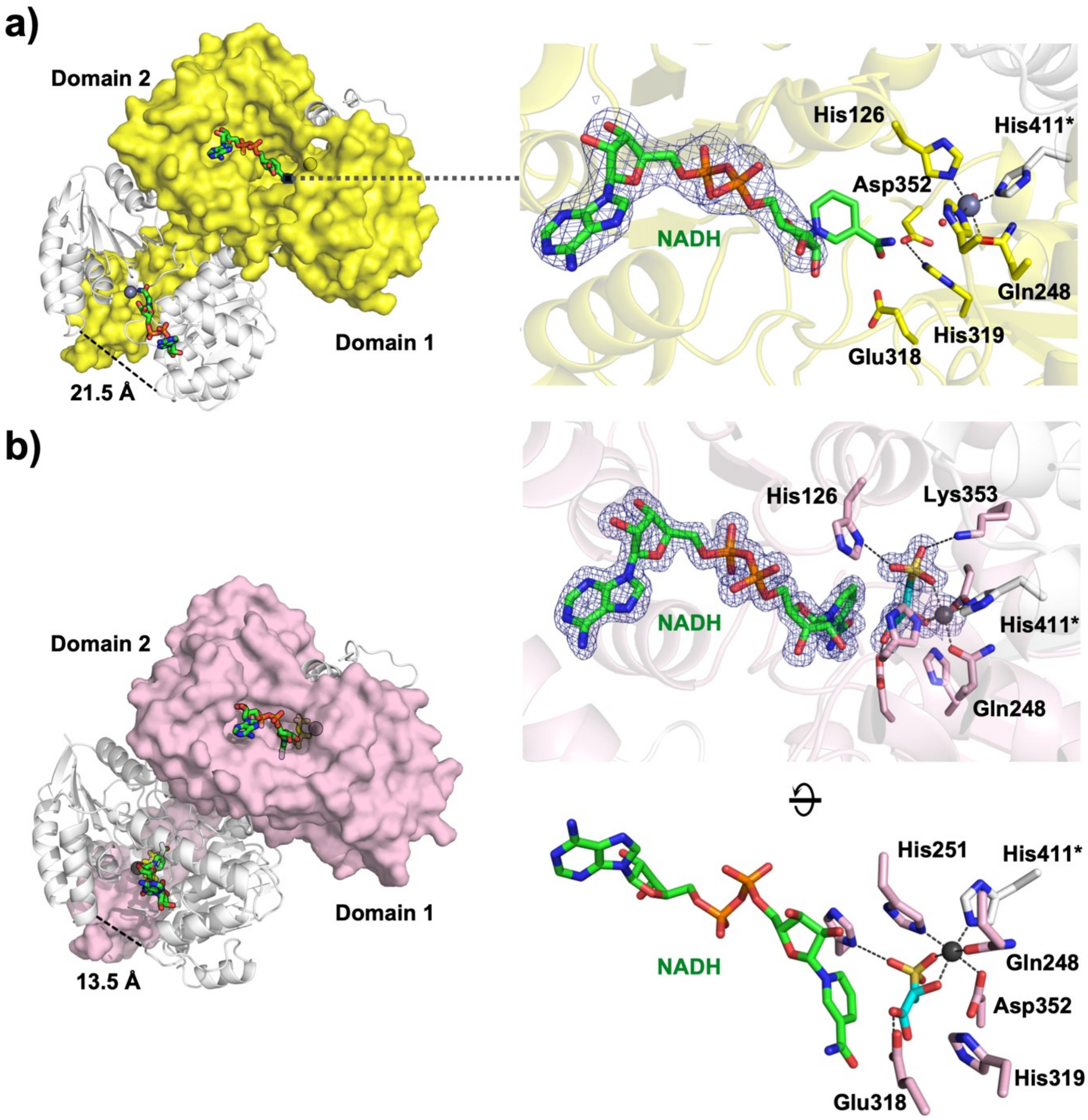

Figure S9. Cofactor and ligand binding modes in *Cp*HpsN. **a**) Crystal structure of *Cp*HpsN in complex with NADH obtained by soaking NADH into *Cp*HpsN•Zn^{2+} crystal. **b**) Co-crystal structure of *Cp*HpsN in complex with *R*-SL and NADH (*Cp*HpsN•Zn^{2+}•NADH•*R*-SL structure). Electron density maps shown in blue mesh are $2F_o - F_c$ maps contoured at 1 σ. The Cα-Cα distance between the tips of domains 1 and 2 (residues 153 and 284, respectively) are displayed, demonstrating the closed conformation in the ternary complex.

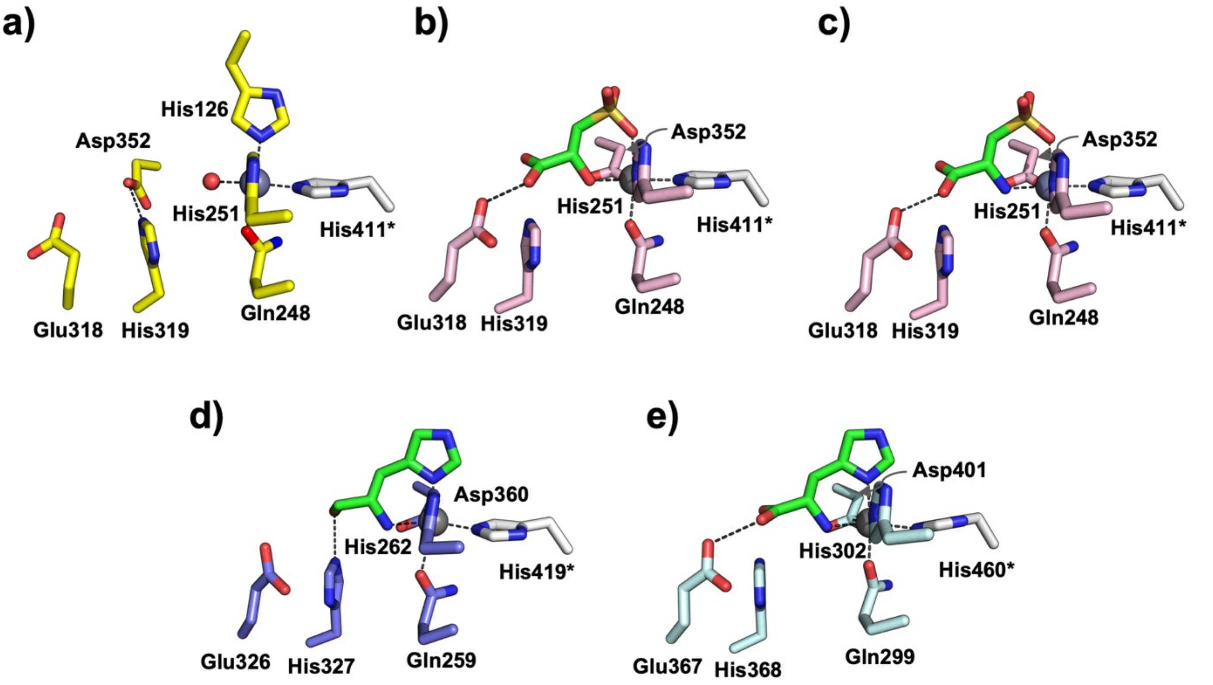

Figure S10. Comparison of the Zn centers of *Cp*HpsN with HisD from *E. coli* and *M. truncatula*. a) Zn^{2+}-loaded apo-*Cp*HpsN, b) *Cp*HpsN•*R*-SL NADH ternary complex, c) *Cp*HpsN•L-cysteate•NADH ternary complex, d) *Ec*HisD•histidinol•NAD^+ ternary complex (PDB entry 1KAE), and e) *Mt*HisD•histidine•NAD^+ ternary complex (PDB entry 5VLD)

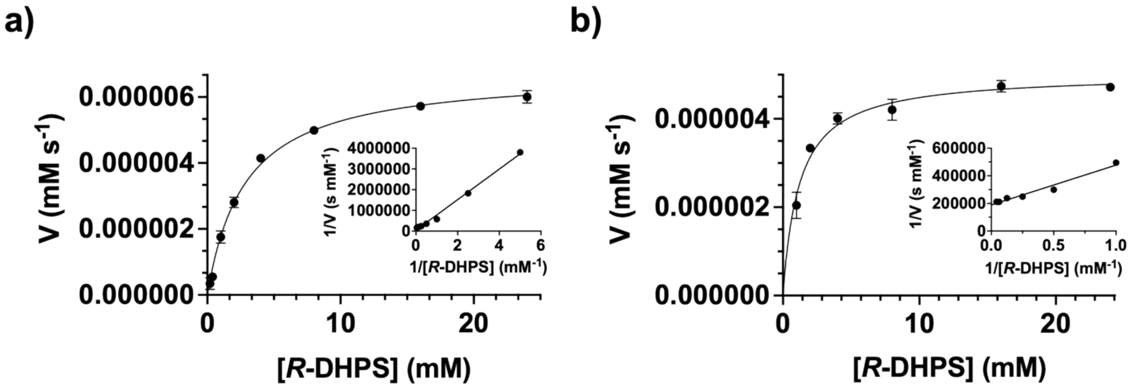

Figure S11. Kinetic analysis of *Cp*HpsN E318A and H319A variants. a,b) Michaelis-Menten and Lineweaver-Burk (inset) plots for reduction of NAD^+ to NADH (oxidation of *R*-DHPS to *R*-SL) with **a**) E318A variant under pseudo first-order conditions of [NAD^+] = 0.30 mM and [*Cp*HpsN-E318A] = 1.34 μM, and **b**) H319A variant under pseudo first-order conditions of [NAD^+] = 0.30 mM and [*Cp*HpsN-H319A] = 1.34 μM. All data is the mean of reaction rates (performed in triplicate). Error bars show standard error mean.

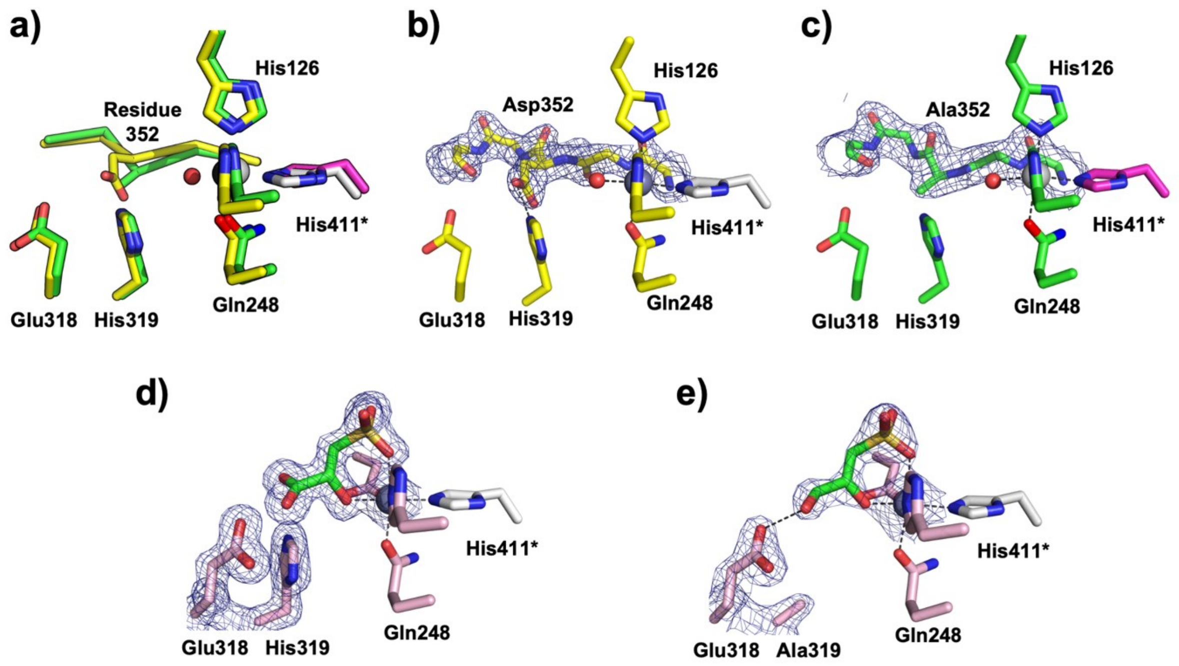

Figure S12. Comparison of 3D structures of mutant *Cp*HpsN with wildtype. a) The superposition of the Zn-center coordinating residues, and proposed catalytic residues. Carbon atoms from chains A and B of wildtype are colored in yellow and light grey, respectively, while those of the D352A variant are in green and magenta, respectively. The backbone Cα positions of residues 350 to 354 are shown in ribbon presentation to display a small change in the Cα position of residue 352 when mutated. The positions of Zn center residues and the two catalytic residues, Glu318 and His319, are almost identical. Zn^{2+} shown in grey spheres (darker sphere for wildtype and lighter sphere for D352A variant). **b-c**) Electron density maps for residues 350–354 in *Cp*HpsN WT (b) and in D352A variant (c), demonstrating the mutation of Asp352 to Ala. **d-e**) Co-crystal structures of WT (d) and H319A variant with the electron density maps for bound ligand (*R*-SL in d and *R*-DHPS in e) and the proposed catalytic residues, demonstrating the difference in residue 319 (His to Ala). *R*-DHPS and Zn^{2+} in the *Cp*HpsN H319A complex are modelled with the occupancy of 0.7. Electron density maps shown in blue mesh are $2F_o - F_c$ maps contoured at 1 σ.

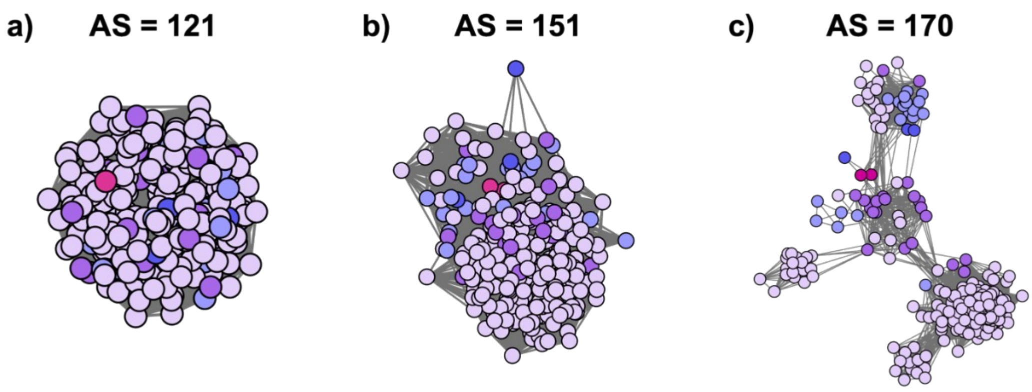

Figure S13. Sequence similarity network (SSN) of HpsN homologues at different alignment scores. a) SSN at minimum alignment score 121, **b**) SSN at minimum alignment score 151, **c**) SSN at minimum alignment score 170. Nodes are colored based on the taxonomic class.

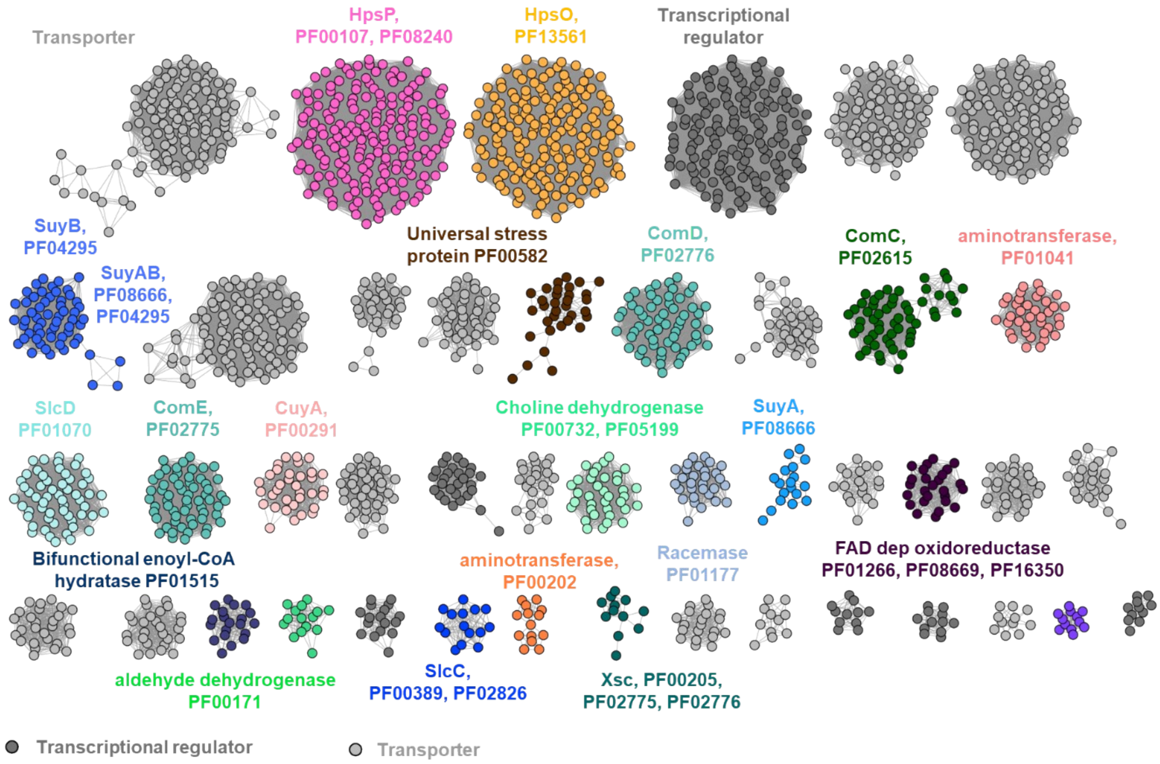

Figure S14. Sequence similarity network of neighbors for HpsN homologues at alignment score 50. Isofunctional clusters are colored according to their annotated function, with protein family codes listed.

Table S1. Diffraction data and refinement statistics.[a]

Data collection	WT	WT•NADH	D352A•NAD^+	WT•NADH•*R*-SL	WT•NADH •L-cysteate	H319A•NADH •*R*-DHPS
Space group	$P2_12_12_1$	$P2_12_12_1$	$P2_12_12_1$	$P2_1$	$P2_1$	$P2_1$
Unit cell parameters (Å)	77.0, 87.8, 134.8	77.3, 87.6, 134.2	76.8, 88.4, 134.1	83.1, 75.6, 85.0 β = 117.3°	82.9, 76.3, 85.1 β = 117.0°	83.3, 75.8, 84.7 β = 117.1°
Resolution (Å)	44.93–1.94 (1.98–1.94)	44.73–2.24 (2.31–2.24)	44.70–2.23 (2.30–2.23)	41.51–1.57 (1.60–1.57)	43.86–1.75 (1.78–1.75)	43.82–2.01 (2.06–2.01)
No. of observations	462,992 (31,038)	300,194 (26,884)	305,583 (27,641)	495,115 (23,030)	274,249 (12,731)	239,908 (16,524)
No. of unique reflections	68,501 (4,501)	44,738 (3,950)	45,320 (4,057)	129,685 (6,020)	94,390 (4,448)	62,003 (4,296)
Completeness (%)	99.9 (98.8)	99.7 (97.4)	99.8 (98.2)	99.4 (93.4)	99.2 (94.8)	99.4 (93.0)
Redundancy	6.8 (6.9)	6.7 (6.8)	6.7 (6.8)	3.8 (3.8)	2.9 (2.9)	3.9 (3.8)
R_{merge} (%)	3.6 (107.3)	4.9 (113.9)	8.6 (119.5)	3.0 (29.6)	3.7 (40.0)	8.2 (69.1)
R_{pim} (%)	1.6 (47.6)	2.2 (51.2)	3.9 (53.3)	2.1 (20.0)	3.2 (32.2)	5.6 (46.3)
$CC_{1/2}$	1.00 (0.79)	1.00 (0.75)	1.00 (0.62)	1.00 (0.90)	1.00 (0.74)	1.00 (0.61)
Average $I/\sigma(I)$	15.5 (1.1)	12.7 (1.1)	10.4 (1.2)	13.9 (2.4)	10.6 (1.7)	7.6 (1.2)
Refinement						
R (%)	21.9 (34.9)	22.3 (36.3)	21.2 (33.0)	14.9 (19.8)	14.8 (21.9)	16.7 (26.1)
R_{free} (%)	26.2 (36.0)	28.0 (37.4)	25.8 (30.8)	17.6 (21.1)	17.7 (25.7)	19.4 (25.4)
No. (%) of reflections in test set	3,480 (5.1)	2,261 (5.1)	2,302 (5.1)	6,496 (5.0)	4,773 (5.1)	2,921 (4.7)
No. of protein molecules per asu	2	2	2	2	2	2
R.m.s.d. bond length (Å)	0.003	0.003	0.003	0.007	0.007	0.004
R.m.s.d. bond angle (°)	0.988	0.917	0.856	1.543	1.534	1.245
Average *B*-factors ($Å^2$)[b]	59.8	74.5	55.1	19.7	22.4	29.2
Protein molecules	55.8 (chain A), 64.6 (chain B)	70.8 (chain A), 78.8 (chain B)	53.9 (chain A), 56.5 (chain B)	20.8 (chain A), 22.1 (chain B)	23.2 (chain A), 25.1 (chain B)	30.7 (chain A), 31.4 (chain B)

Ligands						
Zn^{2+}	45.0 (chain A), 54.1 (chain B)	59.5 (chain A), 68.0 (chain B)	38.5 (chain A), 45.6 (chain B)	14.5(chain A), 16.5 (chain B)	17.0 (chain A), 19.1 (chain B)	31.3 (chain A), 35.3 (chain B)
cofactor (NAD^+ or NADH)	-	69.6 (chain A), 85.7 (chain B)	82.2 (chain A)	16.7 (chain A), 19.4 (chain B)	19.0 (chain A), 22.1 (chain B)	28.0 (chain A), 28.3 (chain B)
Substrate/product/inhibitor	-	-	-	14.1 (chain A), 15.8 (chain B)	16.7 (chain A), 18.9 (chain B)	28.0 (chain A), 30.5 (chain B)
Water molecules	53.7	55.8	45.4	31.7	33.0	36.6
Ramachandran plot[c]						
Residues other than Gly and Pro in:						
Most favoured regions (%)	97.4	96.2	97.1	97.1	97.1	97.0
Additionally allowed regions (%)	2.2	3.8	2.8	2.9	2.9	3.0
Outliers (%)	0.4	0	0.1	0.0	0.0	0.0
**PDB entry code**	**8V35**	**8V36**	**8V37**	**9CP8**	**9CP7**	**9CP9**

[a] Values in parentheses are for the highest-resolution shell.
[b] Calculated by BAVERAGE in CCP4 Suite.[3]
[c] Calculated using MolProbity.[4]

Experimental

1. Reagents

R- and *S*-DHPS were synthesized as described in Burchill *et al.*[5] Racemic SLA was synthesized as described in Abayakoon *et al.*[6] The sodium salt of *R*-SL was synthesized from dicyclohexylammonium sulfolactate[7] by passage through a column of Dowex-50 (Na^+ form). All other reagents and buffers were purchased from Sigma-Aldrich and used as received.

2. Protein Production

Cloning, protein expression and purification of *Cupriavidus pinatubonensis* HpsN

The codon-harmonized *Cp*HpsN gene (WP_011295860.1) was synthesized and cloned between the *Bam*HI and *Eco*RI restriction sites in pRSFDuet-1, with an engineered TEV protease recognition site between the N-terminal hexa-histidine (His_6) tag and *Cp*HpsN. For protein production, pRSFDuet1-*Cp*HpsN was transformed and expressed in *E. coli* BL21(DE3). The cells were grown in lysogeny broth with 50 µg/ml kanamycin at 37 °C and induced with 0.5 mM isopropyl 1-thio-β-D-galactopyranoside (IPTG) when they reached an A_{600} of 0.8. After incubation at 18 °C for 16 h, cells were harvested by centrifugation at 6000 × *g*. The cell pellet was resuspended in buffer A (20 mM Tris ˙ HCl (pH 7.5), 500 mM NaCl, 25 mM imidazole), disrupted using a TS Series benchtop cell-disruptor, and clarified by centrifugation at 40000 × *g*. The soluble fraction was applied to a chelating column (Hi-Trap HP, Cytiva) charged with Ni^{2+}. *Cp*HpsN was eluted using a gradient of 25–500 mM imidazole in buffer A. Pooled fractions were subject to tobacco etch virus (TEV) protease treatment [1:20 (w:w)] in dialysis tubing (10 kDa MWCO) and dialyzed against buffer B (20 mM Tris ˙ HCl (pH 7.5), 200 mM NaCl) for 2 h at room temperature. To avoid potential Ni^{2+} contamination during immobilized metal affinity chromatography, EDTA was added to the dialysis buffer to a final concentration of 1 mM and further dialysis was performed at 4 °C for 16 h. Dialyzed protein samples were concentrated and applied to a size-exclusion column (HiLoad 16/600 Superdex 200 pg) equilibrated with 20 mM Tris ˙ HCl (at pH 7.5), 200 mM NaCl. *Cp*HpsN was reconstituted with 5-fold molar excess of $ZnCl_2$. Final protein samples were concentrated to 5–15 mg/ml, snap-frozen using liquid nitrogen and stored at − 80 °C. For demetallated *Cp*HpsN used for metal screening, the protein was concentrated without $ZnCl_2$ before being snap-frozen and stored at − 80 °C.

Mass photometry

The solution mass of *Cp*HpsN was determined using a mass photometer, TwoMP (Refeyn). The mass calibration was performed using bovine serum albumin (66 kDa monomer and 132 kDa dimer in equilibrium) and thyroglobulin (330 kDa) as standards prior to data acquisition. 10 µl of 100 nM *Cp*HpsN solution was diluted two-fold with 10 µl of protein buffer (20 mM Tris.HCl (pH 7.5), 0.2 M NaCl) pre-loaded on a glass slide, followed by one minute video recording using the AcquireMP

software (Refeyn). Data were analysed with the DiscoverMP software (Refeyn), converting ratiometric contrast values to molecular masses.

Site directed mutagenesis

The plasmid constructs for *Cp*HpsN-E318A, *Cp*HpsN-H319A, and *Cp*HpsN-D352A, were generated with a Q5 site-directed mutagenesis kit (New England Biolabs) using the primers (lower-case letters indicate the mutated sequence):

for E318A: 5'-gcgCACCTGGAAGTGCAC-3' and 5'- GCTCGCATAACGATCGC-3'
for H319A: 5'-gcgCTGGAAGTGCACACACC-3' and 5'- CTCGCTCGCATAACGATC-3'
for D352A: 5'- gcgAAAACCAGCGGTCCG-3' and 5'- ACCAAACGCAACGGTG-3'

Mutagenesis was verified by DNA sequencing and the proteins were expressed and purified by the same method used for the wild-type protein.

3. HPLC-MS conditions for monitoring product formation:

SL formation was monitored by high-performance liquid chromatography-electrospray ionization tandem mass spectrometry (HPLC-ESI-MS/MS) analysis. This used a triple quadrupole mass spectrometer (Agilent 6460 QQQ) coupled with an Agilent 1260 Infinity Series LC system. A ZIC-HILIC column (5 µm, 50 × 2.1 mm; Merck) was used. The HPLC conditions involved a gradient from 90% solvent B to 10% solvent B over a duration of 12 min, followed by 2 min hold at 10% solvent B, and then a return to 90% solvent B over 2 min. Solvent A was 100 mM ammonium acetate in 1% acetonitrile, and Solvent B was 100% acetonitrile. The flow rate was maintained at 0.30 ml/min, and the injection volume was 5 µL. The mass spectrometer was operated in negative ionization mode. For the analysis, the MS/MS multiple reaction monitoring (MRM) mode of the Agilent Mass Hunter Quantitative Analysis software was used. Prior to analysis, the sensitivity for each MRM-MS/MS transition was individually optimized for every analyte. The retention times and ESI-MS/MS fragmentation patterns of the analytes were:

SL: retention time, 8.8 min; ESI-MS/MS *m/z* of $[M-H]^-$ 168.98; product ions 71.2, 151.1.
DHPS: retention time, 6.69 min; ESI-MS/MS *m/z* of $[M-H]^-$ 155.00; product ions 80.2, 95.1.
SLA: retention time, 6.2 min; ESI-MS/MS *m/z* of $[M-H]^-$ 153; product ion 81.

4. Enzyme kinetics

General

Phosphate buffer was not used in our investigations of *Cp*HpsN as it has been shown that NADH is unstable in phosphate buffer.[8] Sulfonate buffers were also avoided due to possible interactions within

the sulfonate binding pocket of *Cp*HpsN. Instead, we opted to employ the wide pH range buffer: Bis-Tris propane. The production of NAD(P)H from oxidation of $NAD(P)^+$ was monitored using a UV-visible spectrophotometer at 340 nm. The extinction coefficient used for NADH was 6363 M^{-1} cm^{-1}.

pH optimization

pH optimization of *R*-DHPS oxidation catalyzed by *Cp*HpsN: Reactions were conducted at 20 °C and contained 100 mM Bis-Tris propane buffer (at varying pH), [NaCl] = 200 mM, [*R*-DHPS] = 0.2 mM, [NAD^+] = 0.3 mM and [$ZnCl_2$] = 50 μM. Reactions were initiated by addition of enzyme to a final concentration, [*Cp*HpsN] = 1.67 μM. Reaction rates were plotted against pH (**Figure S2**). The highest reaction rate was at pH 8.0, which was used for all further kinetic work.

Michaelis-Menten kinetic analysis of *Cp*HpsN

Michaelis-Menten kinetic analysis were performed for racemic SLA, *R*-DHPS, *S*-DHPS, glycerol-3-phosphate (G3P), L-histidinol, NAD^+ and $NADP^+$ under pseudo first-order conditions in which the concentration of one substrate was varied while the other was held constant, and *vice versa* (**Figure 3**). All reactions were performed in triplicate in 100 mM Bis-Tris propane buffer (at pH = 8.0), with [NaCl] = 200 mM and [$ZnCl_2$] = 50 μM. Error bars show standard error mean.

For Michaelis-Menten kinetics of *R*-DHPS with constant NAD^+, reactions were conducted with [*Cp*HpsN] = 67.2 nM, constant [NAD^+] = 0.3 mM, and varying concentrations of *R*-DHPS (0.05–16.0 mM) (**Figure 3b**).

For Michaelis-Menten kinetics of NAD^+ with constant *R*-DHPS, reactions were conducted with [*Cp*HpsN] = 67.2 nm, constant [*R*-DHPS] = 8.0 mM, and varying concentrations of NAD^+ (0.02–3.0 mM) (**Figure 3c**).

For *S*-DHPS with constant NAD^+, reactions were conducted with [*Cp*HpsN] = 230 nM, constant [NAD^+] = 0.3 mM, and varying concentrations of *S*-DHPS (2.0–16.0 mM) (**Figure 3d**).

For NAD^+ with constant *S*-DHPS, reactions were conducted with [*Cp*HpsN] = 230 nM, constant [*S*-DHPS] = 8.0 mM, and varying concentrations of NAD^+ (0.01–6.0 mM) (**Figure 3e**).

For racemic SLA with constant NAD^+, reactions were conducted with [*Cp*HpsN] = 230 nM, constant [NAD^+] = 0.30 mM, and varying concentrations of racemic SLA (0.01–6.0 mM) (**Figure 3f**).

Comparison of NADH production for the *Cp*HpsN catalyzed reactions of SLA or *R*-DHPS indicates a stereoselective preference for D-SLA. The data in **Figure 3** and **Table 1** have been adjusted to reflect this (is [D-SLA] = [SLA]/2).

The apparent kinetic parameters, k_{cat}^{app}, K_M^{app}, and $(k_{cat}/K_M)^{app}$, were calculated using the Prism 9 software package (GraphPad Scientific Software). No measurable reaction was observed for $NADP^+$ (with *R*-DHPS), G3P (with NAD^+) and L-histidinol (with NAD^+), even when *Cp*HpsN was added at a high concentration, [*Cp*HpsN] = 230 nM.

Comparison of turnover of SLA and *R*-DHPS by *Cp*HpsN

Reactions were carried out at 20 °C in 100 mM Bis-Tris propane buffer (at pH = 8.0), with [NaCl] = 200 mM, [$ZnCl_2$] = 50 μM [NAD^+] = 0.3 mM, and [SLA] = 8.0 mM. The reaction was initiated by adding enzyme to [*Cp*HpsN] = 230 nM. The reaction appeared complete after 3 h. Additional *Cp*HpsN was added to make a final concentration of [*Cp*HpsN] = 460 nM, and the reaction was monitored with no further increase in absorbance. Error is calculated as standard error mean.

Metal-Ion Dependence Test

Reactions were conducted at 20 °C and contained 100 mM Bis-Tris propane buffer, [NaCl] = 200 mM, [*R*-DHPS] = 0.2 mM and [NAD^+] = 0.3 mM. Reactions were initiated by addition of enzyme to a final concentration, [*Cp*HpsN] = 85.7 nM. Reaction rates were measured for:

- *Cp*HpsN (Zn^{2+}-loaded) with no metal and [EDTA] = 50 μM.
- *Cp*HpsN (demetallated) with the divalent metal cations: Zn^{2+}, Ba^{2+}, Ca^{2+}, Co^{2+}, Cu^{2+}, Mg^{2+}, Mn^{2+}, Ni^{2+} at 50 μM, or [EDTA] = 50 μM.

L-Cysteate Inhibition Plot

A series of reactions of [*R*-DHPS] = 1.0 mM with [NAD^+] = 0.3 mM, conducted in 100 mM Bis-Tris Propane buffer, [NaCl] = 200 mM, [$ZnCl_2$] = 50 μM at 20 °C were performed with varying concentrations of the inhibitor L-cysteate. Reactions were initiated by *Cp*HpsN at 67.2 nM and performed in triplicate and error bars show SEM. The IC_{50} value of L-cysteate was calculated to be 2.35 ± 0.69 mM (**Figure S5**).

Michaelis-Menten kinetic analysis of *Cp*HpsN variants

Michaelis-Menten kinetic analysis were performed for *R*-DHPS with E318A, H319A and D352A variants of *Cp*HpsN, under pseudo first-order conditions in which the concentration of *R*-DHPS was varied while NAD^+ was held constant (**Figure S5**). Reactions were performed in triplicate at 20 °C using 100 mM Bis-Tris propane buffer (at pH = 8.0), with [NaCl] = 200 mM and [$ZnCl_2$] = 50 μM. Error bars show standard error mean.

For *Cp*HpsN-E318A: Reactions were conducted with [*Cp*HpsN-E318A] = 1.34 μM, constant [NAD^+] = 0.3 mM and varying concentrations of *R*-DHPS (0.05–16.0 mM).

For *Cp*HpsN-H319A: Reactions were conducted with variant enzyme, [*Cp*HpsN-H319A] = 1.34 μM, constant [NAD^+] = 0.3 mM and varying concentrations of *R*-DHPS (0.05–16.0 mM).

For *Cp*HpsN-D352A: No measurable reaction was observed with *R*-DHPS and NAD^+, even at very high concentration, [*Cp*HpsN-D352A] = 97.1 μM.

The apparent kinetic parameters, k_{cat}^{app}, K_M^{app}, and $(k_{cat}/K_M)^{app}$, were calculated using the Prism 9 software package (GraphPad Scientific Software) (**Table 2**).

5. 3D structures of *Cp*HpsN

Protein crystallization and X-ray diffraction data collection

Crystals of apo *Cp*HpsN wild-type and D532A variant (both containing Zn^{2+}) in space group $P2_12_12_1$ were grown by hanging-drop vapor diffusion at 20 °C. 2 µl of protein (5 mg/ml) was mixed with an equal volume of reservoir solution (0.1 M HEPES (at pH 7.5), 20 mM $MgCl_2$, 16–22% (w/v) PEG4000) and equilibrated against 0.5 ml of the reservoir solution. The *Cp*HpsN•NADH and *Cp*HpsN-D352A•NAD^+ complexes were obtained by soaking Zn^{2+} containing apo crystals with 5 mM NADH and 5 mM NAD^+, respectively, for 2 d. Crystals of *Cp*HpsN wild-type and H319A variant in complex with cofactor (NADH) and substrate (*R*-DHPS)/product (*R*-SL)/inhibitor (L-cysteate, L-CSA) in space group $P2_1$ were crystallized from the protein preincubated with 5 mM NADH and 10–16 mM ligand at 4 °C for 30 min. The reservoir solution was composed of 0.1 M Bis-Tris (pH 5.5 – 6.0), 0.2 M $LiSO_4$, 18–28% (w/v) PEG3350.

Crystals were cryoprotected by successive transfer to artificial reservoir solutions containing 20% ethylene glycol in 10% increments at 20 °C, before cryocooled with liquid nitrogen. Diffraction data were recorded on beamline MX2 at the Australian Synchrotron[9] at a wavelength of 0.954 Å at 100 K. The data were processed with XDS[10] and merged and scaled with AIMLESS.[11] Data collection and merging statistics are summarized in **Table S1**.

Crystal structure resolution and refinement

The crystal structure of Zn^{2+}-*Cp*HpsN in space group $P2_12_12_1$ was solved by molecular replacement using PHASER[12] within the CCP4 suite.[3] The AlphaFold[13] predicted model of *Cp*HpsN (AF-Q46N53-F1) was used as initial search model and a solution found with a log likelihood gain of 332 and a Z score of 18.8, locating a dimer in the asymmetric unit. Significant rebuilding around the active site and connecting loops was carried out by iterative model building with COOT,[14] and refinement with REFMAC5.[15] For the complex structures of Zn^{2+}-*Cp*HpsN•NADH and Zn^{2+}-*Cp*HpsN-D352A•NAD^+, the coordinates of the Zn^{2+}-*Cp*HpsN structure were used for the initial refinement after removing all non-protein atoms and setting the temperature factor to 30 $Å^2$.

The structure of *Cp*HpsN in complex with Zn^{2+}, *S*-SL and NADH in space group $P2_1$ was also solved by molecular replacement as described above. A solution was found with a log likelihood gain of 956 and a Z score of 32.9, locating a dimer in the asymmetric unit. For the structures of the *Cp*HpsN•L-CSA•NADH and *Cp*HpsN-H319A•*R*-DHPS•NADH complexes, the coordinates of the *Cp*HpsN•*R*-SL•NADH structure were used for the initial refinement after removing all non-protein atoms and setting the temperature factor to 30 $Å^2$. The quality of the model was validated using MOLPROBITY.[4] The refinement statistics are included in **Table S1**.

6. Bioinformatic analysis

The protein sequence of HpsN from *C. pinatubonensis* (Uniprot accession code: Q46N53) was used as query for BLAST search of UniProt database, which retrieved 1000 sequences with more than 46.2% identity. These sequences were used to generate sequence similarity networks (SSNs) using the Enzyme Function Initiative-Enzyme Similarity Tool (EFI-EST, https://efi.igb.illinois.edu/efi-est/) at different alignment scores i.e., AS = 150, AS = 165, AS = 170 and AS =175 (**Figure S8**). Using the Enzyme Function Initiative-Genome Neighborhood Tool (EFI-GNT, https://efi.igb.illinois.edu/efi-gnt/), clusters within the SSNs were identified, numbered and colored, and the neighborhood PFAM codes (± 10 open reading frame window) were retrieved. The SSN and the PFAMs of proteins encoded in their genome neighborhoods were manually inspected for the presence of both HpsO and HpsP encoded in their genome neighborhood, leading to the selection of 272 sequences assigned as HpsN homologues.

272 sequences were used to generate SSNs at different alignment scores (AS) i.e., AS = 150, AS = 165, AS = 170 and AS =175. The SSN at AS = 170 (corresponding to 62% identity) was used to generate genome neighborhood diagrams (GND) with an open reading frame (ORF) ± 10 neighbors using the EFI-GNT. A script was used to extract the accession codes of the retrieved neighbors. These neighbors accession codes were used to generate a sequence similarity network of neighbors (SSNN) with minimum alignment score of 50. The resulted network (SSNN) was plotted using Cytoscape v3.8[16]. Subsequently, Uniprot BLASTp searches were conducted individually for different sulfolyases i.e., SuyAB, CuyA and Xsc homologues in organisms lacking adjacent genes encoding sulfolyase enzymes and these results were mapped to the SSN for further analysis. The individual nodes of the HpsN SSN (AS = 170) were colored according to occurrence of gene homologues from three different pathways in the genome neighborhood or in the case of the genes encoding SuyAB, CuyA and Xsc, elsewhere in the genome. To display the taxonomic distribution of HpsN, the SSN was manually colored based on the source organism class.

SI References

1. R. A. Laskowski, *Prot. Sci.*, 2022, **31**, e4473.
2. C. S. Bond, *Bioinformatics*, 2003, **19**, 311-312.
3. M. D. Winn, C. C. Ballard, K. D. Cowtan, E. J. Dodson, P. Emsley, P. R. Evans, R. M. Keegan, E. B. Krissinel, A. G. W. Leslie, A. McCoy, S. J. McNicholas, G. N. Murshudov, N. S. Pannu, E. A. Potterton, H. R. Powell, R. J. Read, A. Vagin and K. S. Wilson, *Acta Crystallogr. D*, 2011, **67**, 235-242.
4. C. J. Williams, J. J. Headd, N. W. Moriarty, M. G. Prisant, L. L. Videau, L. N. Deis, V. Verma, D. A. Keedy, B. J. Hintze, V. B. Chen, S. Jain, S. M. Lewis, W. B. Arendall Iii, J. Snoeyink, P. D. Adams, S. C. Lovell, J. S. Richardson and D. C. Richardson, *Protein Sci.*, 2018, **27**, 293-315.
5. L. Burchill, L. Zudich, P. L. van der Peet, J. M. White and S. J. Williams, *J. Org. Chem.*, 2022, **87**, 4333-4342.

6. P. Abayakoon, R. Epa, M. Petricevic, C. Bengt, J. W. Y. Mui, P. L. van der Peet, Y. Zhang, J. P. Lingford, J. M. White, E. D. Goddard-Borger and S. J. Williams, *J. Org. Chem.*, 2019, **84**, 2901-2910.
7. D. E. Graham, H. Xu and R. H. White, *J. Biol. Chem.*, 2002, **277**, 13421-13429.
8. E. Aamer, J. Thöming, M. Baune, N. Reimer, R. Dringen, M. Romero and I. Bösing, *Sci. Rep.*, 2022, **12**, 16380.
9. D. Aragao, J. Aishima, H. Cherukuvada, R. Clarken, M. Clift, N. P. Cowieson, D. J. Ericsson, C. L. Gee, S. Macedo, N. Mudie, S. Panjikar, J. R. Price, A. Riboldi-Tunnicliffe, R. Rostan, R. Williamson and T. T. Caradoc-Davies, *J. Synchrotron Radiation*, 2018, **25**, 885-891.
10. W. Kabsch, *Acta Crystallogr., Section D: Biol. Crystallogr.*, 2010, **66**, 125-132.
11. P. R. Evans and G. N. Murshudov, *Acta Crystallogr. Sect. D*, 2013, **69**, 1204-1214.
12. A. J. McCoy, R. W. Grosse-Kunstleve, P. D. Adams, M. D. Winn, L. C. Storoni and R. J. Read, *J. Appl. Crystallogr.*, 2007, **40**, 658-674.
13. J. Jumper, R. Evans, A. Pritzel, T. Green, M. Figurnov, O. Ronneberger, K. Tunyasuvunakool, R. Bates, A. Žídek, A. Potapenko, A. Bridgland, C. Meyer, S. A. A. Kohl, A. J. Ballard, A. Cowie, B. Romera-Paredes, S. Nikolov, R. Jain, J. Adler, T. Back, S. Petersen, D. Reiman, E. Clancy, M. Zielinski, M. Steinegger, M. Pacholska, T. Berghammer, S. Bodenstein, D. Silver, O. Vinyals, A. W. Senior, K. Kavukcuoglu, P. Kohli and D. Hassabis, *Nature*, 2021, **596**, 583-589.
14. P. Emsley, B. Lohkamp, W. G. Scott and K. Cowtan, *Acta Crystallogr. Sect. D*, 2010, **66**, 486-501.
15. M. D. Winn, G. N. Murshudov and M. Z. Papiz, *Methods Enzymol.*, 2003, **374**, 300-321.
16. P. Shannon, A. Markiel, O. Ozier, N. S. Baliga, J. T. Wang, D. Ramage, N. Amin, B. Schwikowski and T. Ideker, *Genome Res.*, 2003, **13**, 2498-2504.
